# Mercury Removal from Contaminated Water by Wood-Based Biochar Depends on Natural Organic Matter and Ionic Composition

**DOI:** 10.1021/acs.est.2c01554

**Published:** 2022-08-04

**Authors:** Sampriti Chaudhuri, Gabriel Sigmund, Sharon E. Bone, Naresh Kumar, Thilo Hofmann

**Affiliations:** † Department of Environmental Geosciences, Centre for Microbiology and Environmental Systems Science, University of Vienna, Josef-Holaubek-Platz 2, Vienna 1090, Austria; ‡ Doctoral School in Microbiology and Environmental Science, University of Vienna, Josef-Holaubek-Platz 2, Vienna 1090, Austria; § Stanford Synchrotron Radiation Lightsource, SLAC National Accelerator Laboratory, 2575 Sand Hill Road, Menlo Park, California 94025, United States; ∥ Soil Chemistry and Chemical Soil Quality Group, Wageningen University, Wageningen 6708 PB, The Netherlands

**Keywords:** sorption, speciation, industrial effluents, porosity, cation bridge, ligand exchange, extended X-ray absorption spectroscopy, reduction

## Abstract

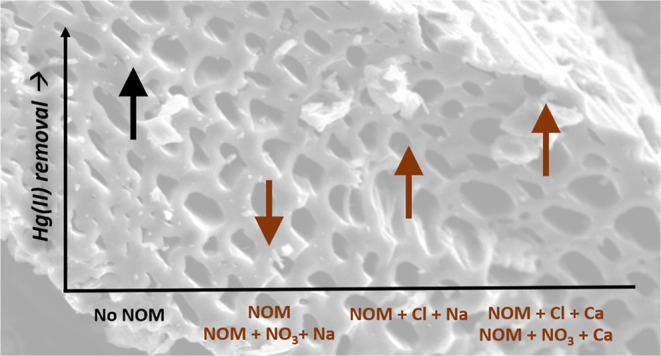

Biochars can remove potentially toxic elements, such as inorganic mercury [Hg(II)] from contaminated waters. However, their performance in complex water matrices is rarely investigated, and the combined roles of natural organic matter (NOM) and ionic composition in the removal of Hg(II) by biochar remain unclear. Here, we investigate the influence of NOM and major ions such as chloride (Cl^–^), nitrate (NO_3_^–^), calcium (Ca^2+^), and sodium (Na^+^) on Hg(II) removal by a wood-based biochar (SWP700). Multiple sorption sites containing sulfur (S) were located within the porous SWP700. In the absence of NOM, Hg(II) removal was driven by these sites. Ca^2+^ bridging was important in enhancing removal of negatively charged Hg(II)-chloro complexes. In the presence of NOM, formation of soluble Hg-NOM complexes (as seen from speciation calculations), which have limited access to biochar pores, suppressed Hg(II) removal, but Cl^–^ and Ca^2+^ could still facilitate it. The ability of Ca^2+^ to aggregate NOM, including Hg-NOM complexes, promoted Hg(II) removal from the dissolved fraction (<0.45 μm). Hg(II) removal in the presence of Cl^–^ followed a stepwise mechanism. Weakly bound oxygen functional groups in NOM were outcompeted by Cl^–^, forming smaller-sized Hg(II)-chloro complexes, which could access additional intraparticle sorption sites. Therein, Cl^–^ was outcompeted by S, which finally immobilized Hg(II) in SWP700 as confirmed by extended X-ray absorption fine structure spectroscopy. We conclude that in NOM containing oxic waters, with relatively high molar ratios of Cl^–^: NOM and Ca^2+^: NOM, Hg(II) removal can still be effective with SWP700.

## Abstract 2

Water chemistry strongly changes Hg(II) removal by biochar via several mechanisms. These changes are driven by combined effects of organic matter and major ions.

## Introduction

The prevalence and acute toxicity of mercury (Hg(II)) have been well documented.^1^ In 2013, the Minamata Convention recognized the immediate need to reduce the use of Hg(II) and Hg emissions globally.^2^ Despite efforts to limit Hg(II) waste, anthropogenic activities generating Hg(II)-containing wastewaters continue in many developing nations. For example, effluents from many gold mining areas,^3^ coal power plants,^4^ biomedical,^5^ paper and pulp industries,^6^ and even groundwater in some historically contaminated sites^7^ still continue to report high Hg(II) concentrations ranging between 0.75 and 10 μM. To treat such contaminated waters, novel materials are being developed, including polymers,^8^ functionalized clays,^9^ carbonaceous materials,^10^ and composites thereof.^11^ Although these sorbents are widely investigated, their large-scale application may be economically challenging in low-income countries. The use of a low-cost carbonaceous sorbent such as biochar may provide an alternative to treat Hg(II)-contaminated waters. Biochar can be produced from agricultural and wood waste, which can support sustainable remediation approaches.

Studies report efficient removal of Hg(II) (as ionic Hg^2+^) using biochar due to its high porosity, surface functional groups that complex with Hg(II), and its highly aromatic structure enabling Hg−π binding.^12,13^ However, Hg^2+^ is a rare form of Hg(II) in water and occurs only under strongly acidic environments and in the absence of complexing ligands.^14^ In contaminated waters, Hg(II) removal efficiency in sulfide-free conditions will be dependent on pH, natural organic matter (NOM) content, and ions such as chloride (Cl^–^), which determine Hg(II) speciation as HgCl_2_, HgCl_3_^1–^, HgCl_4_^2–^, HgClOH, or Hg-NOM complexes.

NOM, being ubiquitous in contaminated waters,^15^ may inhibit Hg(II) removal due to the formation of aqueous Hg-NOM complexes and competition over sorption sites.^16,17^ Sorbed NOM can also offer new sorption sites and increase Hg(II) removal from aqueous solutions.^18^ Cl^–^ is a major anion in many natural and anthropogenic waters^19^ and strongly complexes with Hg(II) as Hg(II)-chloro complexes.^20^ Higher Cl^–^ concentrations forming stable dissolved HgCl_2_ can inhibit Hg(II) immobilization.^21^ The ratios of NOM/Hg(II), Cl^–^/Hg(II), and NOM/Cl^–^ determine Hg(II) distribution as Hg-NOM or Hg(II)-chloro complexes. The presence of other cations induces changes in NOM by increasing their hydrophobicity through inner and outer sphere complexation.^22^ This may affect the complexation behavior of Hg(II) with NOM and its removal by sorbents.

Water chemistry also influences the biochar surface: alkaline pH increases its negative surface charge,^23^ NOM can block small pores and sorb onto the surface,^24^ and cations in solution can decrease the negative surface charge of biochar.^25^ Ternary interactions between biochar, Hg(II), and NOM, as well as coexisting ions are expected to impact Hg(II) removal by biochar. These intertwined mechanisms are largely under-investigated. The influence of complex water chemistry on Hg(II) immobilization by novel carbonaceous sorbents such as biochar is crucial to understand prior to full-scale application.

To address this knowledge gap, we aim to investigate the influence of major anions, cations, and NOM on Hg(II) removal by a standardized wood-based biochar under sulfide-free conditions. Complex systems are represented by different coexisting background ions with peat soil-extracted NOM. We investigate the interdependence of these parameters on Hg(II) removal from contaminated waters having high Hg(II) concentrations typical of wastewater effluents and streams. Laboratory batch experiments are combined with X-ray absorption spectroscopy to provide mechanistic insights into the atomic coordination environments of immobilized Hg(II). Although NOM may decrease Hg(II) removal, we hypothesize that the existence of Cl^–^ and Ca^2+^ in such systems can counter this effect. Our study advances the applicability of biochars in removing Hg(II) from contaminated waters with different background complexities.

## Materials and Methods

### Preparation of NOM Extract

NOM was extracted from bulk Pahokee Peat material (IHSS-2BS103P) because it is a well-investigated representative of the complex NOM encountered in field scenarios. NOM was extracted via an alkaline extraction process, detailed in Section 1 of the Supporting Information. This extract was quantified for operationally defined (<0.45 μm) dissolved organic carbon (DOC) on a TOC analyzer (TOC-L_CPH/CPN_, Shimadzu, Japan), measuring nonpurgeable organic carbon, and used as stock for all sorption experiments. A portion of this extract was lyophilized to determine the total C and S contents using an elemental analyzer (Elementar VarioMacro, Elementar Analysensysteme GmbH, Germany). NOM was calculated to be 2.31 times the DOC concentration in mg L^–1^.

### Background Solutions

Deionized water derived from an Elga PURELAB Chorus 3 water purification unit (ELGA LabWater, UK) was used to prepare all solutions. Solutions with different background electrolytes were produced from 1M stock solutions of sodium chloride (NaCl), sodium nitrate (NaNO_3_), calcium chloride dihydrate (CaCl_2_·2H_2_O), and calcium nitrate tetrahydrate [Ca(NO_3_)_2_·4H_2_O]. Hg(II) stock solution was prepared by dissolving mercuric chloride (HgCl_2_) in water. All salts were of analytical grade and purchased from Merck, Germany.

Hg-NOM intermediate working solutions were diluted from stock solutions of Hg(II) and NOM and pre-equilibrated in the dark for 72 h to allow Hg(II) to complex with available binding sites in NOM. Hg(II) reacts with strong binding sites containing S within a few hours.^26^ Since we investigated scenarios of higher Hg(II) contamination, such as waste water streams and industrial effluents, a longer reaction time was allowed to facilitate Hg(II) complexation with O containing sites. Oxic conditions and a preliminary experiment with these solutions ensured that NOM caused minor formation of Hg(0), that is, 93 ± 3% of Hg(II) was recovered from the aqueous phase and the bottle walls, after purging with N_2_ for 30 min.

The solutions used in the sorption experiments were composed of mixtures of equal volumes of Hg(II) intermediate solution (Hg-NOM or NOM-free) and electrolyte solution with the final Hg(II) concentration kept at 2.5 μM. The pH was allowed to equilibrate to a value buffered by the biochar at 6.9 ± 0.6 (mean ± SD). Owing to the range of NOM concentrations in contaminated waters,^27^ we conducted experiments using 0 mg L^–1^ NOM, 4.6 mg L^–1^ NOM, and 46.2 mg L^–1^ NOM with extensive investigation at 46.2 mg L^–1^ where significant effects were discernible. To study the effects of individual ions and their concentrations, we kept the concentration of the respective anionic/cationic counterpart constant. Maximum Ca^2+^ concentration was kept at 20 mM since higher concentrations typically do not occur^27^ and would have aggregated all NOM, reducing the reliability of our results.

A separate set of experiments with NaCl solutions were conducted at a lower pH of 5.0 using 10 mM 2-(*N*-morpholino) ethanesulfonic acid (MES) buffer purchased from Alfa Aesar, Germany. MES buffer has previously been used in studies with NOM,^28^ Hg(II),^29^ and with solid phases including biochar^30^ and soil.^31^

### Windermere Humic Aqueous Model VI Speciation of Background Solutions

Approximate estimations of Hg(II) speciation in each background solution were made using the Windermere Humic Aqueous Model (WHAM VI), which assumes a series of discrete site p*K*_a_ values for metal binding with humic substances. WHAM VI combines the inorganic speciation code WHAM with a submodel of humic ion binding model VI. Oftentimes, metal binding by NOM can be overestimated if the true proportion of NOM available for metal binding is not used.^32^ Therefore, we assumed that the binding sites for Hg(II) exist predominantly in the hydrophobic fraction of NOM (humic and fulvic acids). For NOM-containing systems, we updated the model with lowered intrinsic binding constants of Hg(II) with humic and fulvic acids (assuming these are only carboxylic and phenolic groups) and separate thermodynamic constants for reactive thiol groups. Further details, including the assumptions made, the equations and constants used, and calculations, are provided in Section 2 of the Supporting Information.

### Sorption Experiments

The majority of biochars reported in the literature are produced within 500–800 °C.^33^ A woody biochar produced from soft wood pellets (5:95 pine/spruce) pyrolyzed at 700 °C (SWP700) was purchased from UK Biochar Research Center (UKBRC, Edinburg, UK). We chose this relatively high-temperature biochar based on its good performance observed in preliminary sorption tests among a suite of 10 standardized biochars (data not shown). Higher pyrolysis temperatures may result in the loss of C-bound S groups, which are the principal drivers for Hg(II) removal.^34^ Further details on the biochar production and characterization are provided in Section 4 of the Supporting Information.

For each experiment, 25 mg of crushed SWP700 (<0.25 mm) was equilibrated in the dark at 125 rpm with 25 mL of background solutions containing Hg(II). SWP700 only leached low levels of NOM (2 ± 1.2 mg C L^–1^) and inorganic ions (<0.01 mM of anions and cations measured by ion chromatography and inductively coupled plasma optical emission spectroscopy, respectively) and caused negligible changes to water chemistry during experimentation. After 48 h, the tubes were centrifuged at 1000 g (Sorvall LYNX 6000, Thermo Fischer, USA) and the solutions were filtered through 0.45 μm cellulose acetate filters (Sartorius, Germany). The filtered supernatants were analyzed for total Hg(II) following US EPA method 1631 using cold vapor atomic fluorescence spectroscopy (CVAFS, DMA 80-L, Milestone, Italy), and sorption coefficients (*K*_d_) were calculated. Details on the measurement and calculations are provided in Sections 5 and 6 of the Supporting Information, respectively. Control experiments without biochar were used to check for the aqueous phase recovery of Hg(II) in background solutions. Loss to filter material was monitored and found to be negligible. Details for both are provided in Section 3 of the Supporting Information. Tukey’s post hoc test was applied after running a one-way ANOVA on the *K*_d_ values to examine the differences (*p* < 0.05) between pairs of means. In cases of skewed *K*_d_ observations, because of a *K*_d_ value larger than the rest by 2 orders of magnitude, we ran the tests excluding that value.

The zeta (ζ) potential of the biochar suspensions, which correlates with the surface charge, was calculated by measuring the electrophoretic mobility using a Litesizer 500 (Anton Paar GmbH, Austria) system. Section 7 of the Supporting Information provides further details of the measurements. The DOC in solutions with high NOM (46.2 mg L^–1^ of NOM) was monitored after sorption experiments using a TOC analyzer (TOC-L_CPH/CPN_, Shimadzu, Japan).

### Solid-Phase Analysis

Four selected scenarios at the highest investigated NOM (46.2 mg L^–1^) and electrolyte concentrations [100 mM NaCl, 100 mM NaNO_3_, 20 mM CaCl_2_, and 20 mM Ca(NO_3_)_2_] were scaled up 10-fold (250 mg in 250 mL) at the same Hg(II) concentration for solid-phase analysis. At the end of sorption experiments, the samples were filtered under vacuum. To wash out remaining salts, the solids were resuspended and washed with deionized water multiple times. Last, the samples were dried in a desiccator under vacuum. The final loading of Hg(II) on the biochars was >250 mg kg^–1^ for all, except the NaNO_3_ system (<50 mg kg^–1^).

Hg solid-phase speciation [samples with Hg(II) > 250 mg kg^–1^] was determined using Hg L_III_-edge extended X-ray absorption fine structure (EXAFS) spectroscopy conducted at beamline 7–2 at the Stanford Synchrotron Radiation Lightsource, which is equipped with a Si(111) double-crystal monochromator. Two-dimensional micro-X-ray fluorescence (μ-XRF) mapping using a 25 μm diameter focused beam was initially used to find associations between elements including Hg, Cl, and S. A representative 25 μm spot from each sample was selected for Hg L_III_-edge EXAFS spectroscopy. All data processing and fitting of the EXAFS spectra were performed using Athena and Artemis,^35^ respectively. A detailed description of the μ-XRF mapping, selection of EXAFS points, data processing, and fitting can be found in Sections 10 and 11 of the Supporting Information.

X-ray diffractograms (XRD) were obtained for the vacuum-dried samples to rule out crystalline-phase precipitation during the experiment [for instance reduction of Hg(II) to Hg_2_Cl_2_]. Energy-dispersive X-ray spectroscopy (EDS) was used to detect significant quantities of elements such as Cl, Ca, and Na on the biochar surface after sorption. Details for both measurements are provided in Section 9 of the Supporting Information.

## Results and Discussion

### Microporous SWP700 Removes Hg(II) by Multiple Mechanisms

SWP700 is a woody biochar produced at 700 °C. Due to the high pyrolysis temperature, SWP700 is highly carbonized with 90.0% C, 6.1% O, and a specific surface area of 184.4 m^2^ g^–1^ (Table S4).

**Figure 1:**
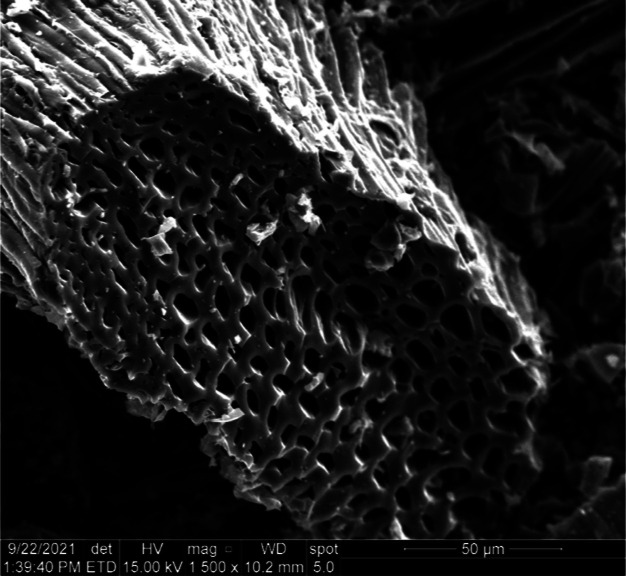
Scanning electron micrograph of SWP700 biochar particle (magnification 1500×).

High pyrolytic temperature in biochars generally translates to high pore volume and water holding capacity.^36^ SEM imaging of SWP700 shows a highly porous framework consisting of macro- (Figures 1 and S2), meso-, and micropores, which could be penetrated by water transporting dissolved Hg(II) species. Recent studies using the same biochar show that 95% of the total porosity in SWP700 corresponds to a pore width of <3 μm,^37^ with 1.4 and 7.8% consisting of micro- (<2 nm) and mesopores (2–50 nm), respectively.^38^ Hg(II) can be immobilized at sites on the external surface and within this porous framework.

The measured low H/C molar ratio of 0.22 indicates a highly condensed aromatic structure that makes SWP700 more aromatic than 92.5% of biochars screened in a recent meta-analysis.^33^ The high aromaticity of SWP700 can cause its π electron system to substantially contribute to Hg(II) immobilization (eq 1).^12,13^
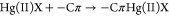
1

At the studied pH of 6.9 ± 0.6, carboxylic moieties on SWP700 are deprotonated and can also participate in complexation reactions (eqs 2 and 3).
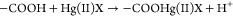
2
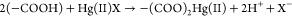
3

Binding constants (log K) of Hg(II) with carboxylic moieties are between 7.3 and 11, while those for Hg(II) with S moieties (e.g., thiols) are higher in the range of 21–47.^49^ Binding with S moieties is thus expected to govern Hg(II)-biochar interactions as long as such sites are available.^39^ With a total S content of 0.18% (Table S4) and assuming reactive *S* of 75% for a high-temperature woody biochar,^40^ 17 mM S binding sites on SWP700 (*S*_reactive,SWP700_) were available per mM Hg(II) at the experimental solid-to-liquid ratio. Thus, S binding sites were in excess of Hg(II) and were expected to drive Hg(II) removal from the aqueous phase (eq 4).
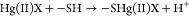
4

### Coexistence of Ca^2+^ and Cl^–^ Influences Hg(II) Removal in the Absence of NOM

**Figure 2:**
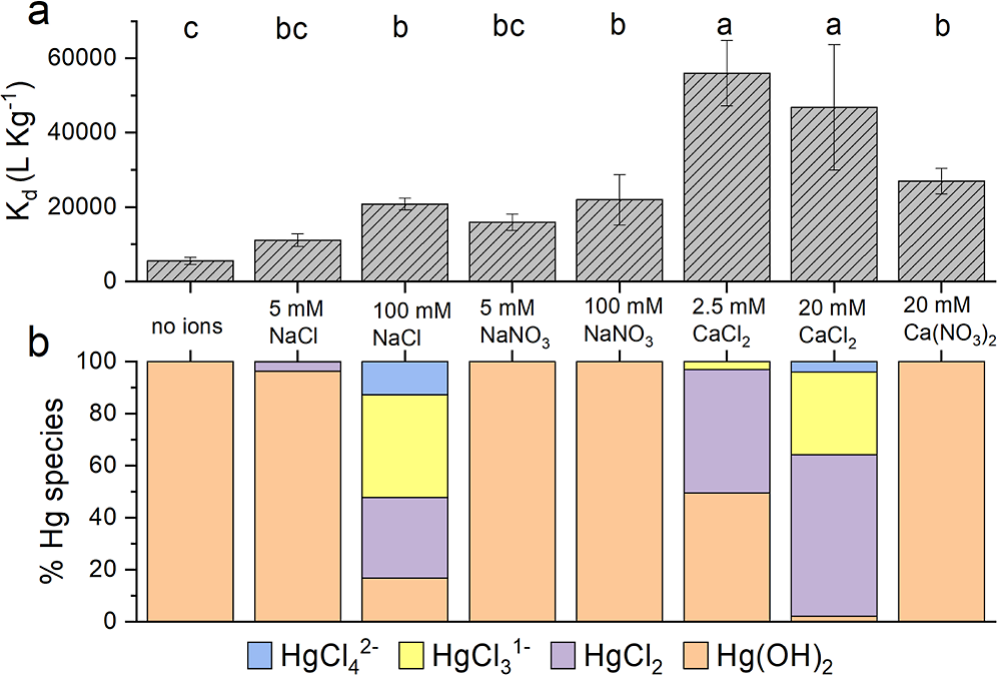
Hg(II) removal by SWP700 without NOM in the presence of different background ions. (a) Sorption coefficients (*K*_d_) of Hg(II), with (b) corresponding Hg(II) speciation in the background solution modeled using the geochemical code WHAM. pH values used in modeling were derived from measurements at the end of the sorption batch experiments, which are shown in Figure S3 of the Supporting Information. Error bars represent standard deviation of at least triplicate measurements. For pairwise comparison of means, the Tukey’s post hoc test was used after running one-way ANOVA (*p* < 0.05).

Figure 2a shows the different *K*_d_ values observed under different water chemistry conditions in NOM-free systems. SWP700 was an effective sorbent for Hg(II), with *K*_d_ ranging between 5500 and 56 000 L kg^–1^. Other studies using similar unmodified biochars and activated carbons made from coconut shell,^13^ bagasse,^13^ pine,^17^ banana peel,^41^ and corn straw^42^ report similar *K*_d_ values ranging between 5000 and 25 000 L kg^–1^. SWP700 was also more effective than two activated carbons we previously tested—a wood-based activated carbon and a commercial powdered activated carbon NORIT Super SAE—which had *K*_d_ values of <1000 and 20 000 L kg^–1^, respectively (data not shown).

WHAM calculations (Figure 2b) indicate that in deionized water and solutions containing either NaNO_3_ or Ca(NO_3_)_2_, Hg(II) exists in the hydroxyl form [as Hg(OH)_2_]. With the addition of 5 mM NaCl, HgCl_2_ is formed and additional Cl^–^ leads to the dominance of negatively charged species (HgCl_4_^2–^ and HgCl_3_^1^). With the exception of CaCl_2_ (*K*_d_ between 46 795 to 56 000 L kg^–1^), significant differences in Hg(II) removal by SWP700 were absent when comparing various ionic compositions (Figure 2a). Lower pH values were observed in SWP700 suspensions with Ca^2+^ (between 6.2 and 6.8) compared to Na^+^ (between 7.5 and 8.0) (Figure S3). This drop in pH can be linked to competition of Ca^2+^ with protons for proton binding sites in SWP700. The electro-kinetic potential (ζ-potential) is often used as a proxy for particle surface charge. Substantially decreased negative ζ-potentials of SWP700 indicate a higher degree of charge screening by Ca^2+^ (−11 to −21 mV) compared to Na^+^ (−39 to −53.5 mV). In CaCl_2_ systems, this facilitates the sorption of negatively charged Hg(II)-chloro complexes, and cation bridging might have further enhanced this removal.^43^ Furthermore, we observed distinctively lower Hg(II) removal (p < 0.05) in Ca(NO_3_)_2_ systems where charged Hg(II)-chloro species were absent. The speciation of Hg(II) as charged Hg(II)-chloro molecules and the coexistence of divalent cations are important to understand enhanced Hg(II) removal in systems without NOM.

### Increasing NOM Concentrations Decreases Hg(II) Removal

**Figure 3:**
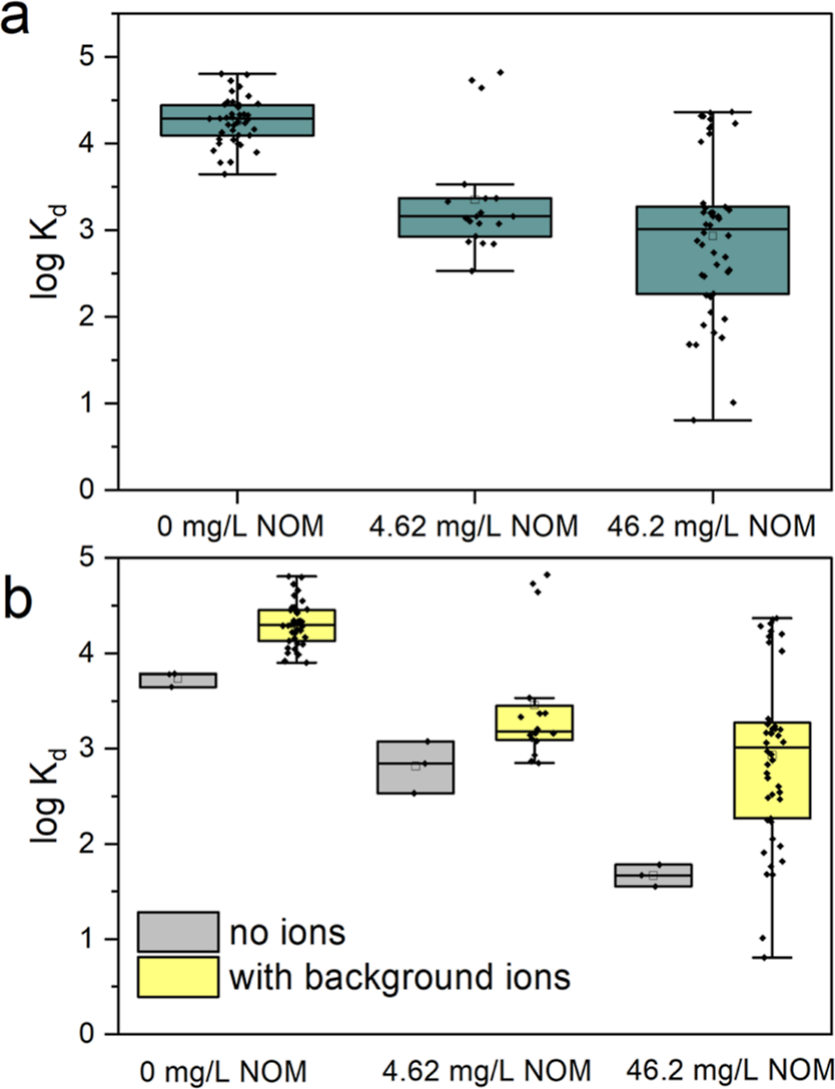
Trends showing Hg(II) removal in the presence of NOM. (a) Box plots of logarithmic sorption coefficients across different NOM levels and (b) comparison of box plots of logarithmic sorption coefficients across NOM levels without and with background ions (5 mM Na^+^ + 5 mM Cl^–^, 100 mM Na^+^ + 100 mM Cl^–^, 2.5 mM Ca^2+^ + 5 mM Cl^–^, 20 mM Ca^2+^ + 40 mM Cl^–^, 2.5 mM Ca^2+^ + 5 mM and NO_3_^–^, and 20 mM Ca^2+^ + 40 mM NO_3_^–^).

We observed a trend of decreasing Hg(II) removal with increasing NOM concentrations (Figure 3a). Presence of NOM leads to the formation of thermodynamically stable Hg-NOM species. Humic and fulvic acids in Pahokee Peat are large NOM molecules and have relatively high molecular weights (weight-averaged molecular weights of 15.4 kDa and 6.9 kDa, respectively).^44^ Such large NOM molecules complexed to Hg(II) would have limited access to internally located pores in SWP700. SWP700 contains abundant graphitic microstructures (Figure 1) with potential Hg(II) sorption sites. Feng et al.^45^ showed that at pyrolysis temperatures between 300 and 900 °C, S distribution extends to locations within this porous network in woody biochars. S sites within SWP700 may thus be crucial for Hg(II) removal in our study. In addition, NOM molecules can block pores and compete for sorption sites by attaching onto the SWP700’s surface via π–π electron donor–acceptor interactions and hydrophobic effects.^46^ Thus, NOM may have inhibited Hg(II) removal by SWP700 via these two complementary processes.

In deionized water, the logarithmic *K*_d_ at 46.2 mg L^–1^ of NOM was 1.6 (Figure 3b). However, we observed that the presence of other background ions (Cl^–^, NO_3_^–^, Ca^2+^, and Na^+^) caused large variations in *K*_d_ spanning more than 3 orders of magnitude (log *K*_d_ between 0.8 and 4.4). This suggests that in the presence of NOM, Hg(II) removal is also affected by ionic composition.

### Ionic Composition Determined Hg(II) Removal in the Presence of NOM

**Figure 4:**
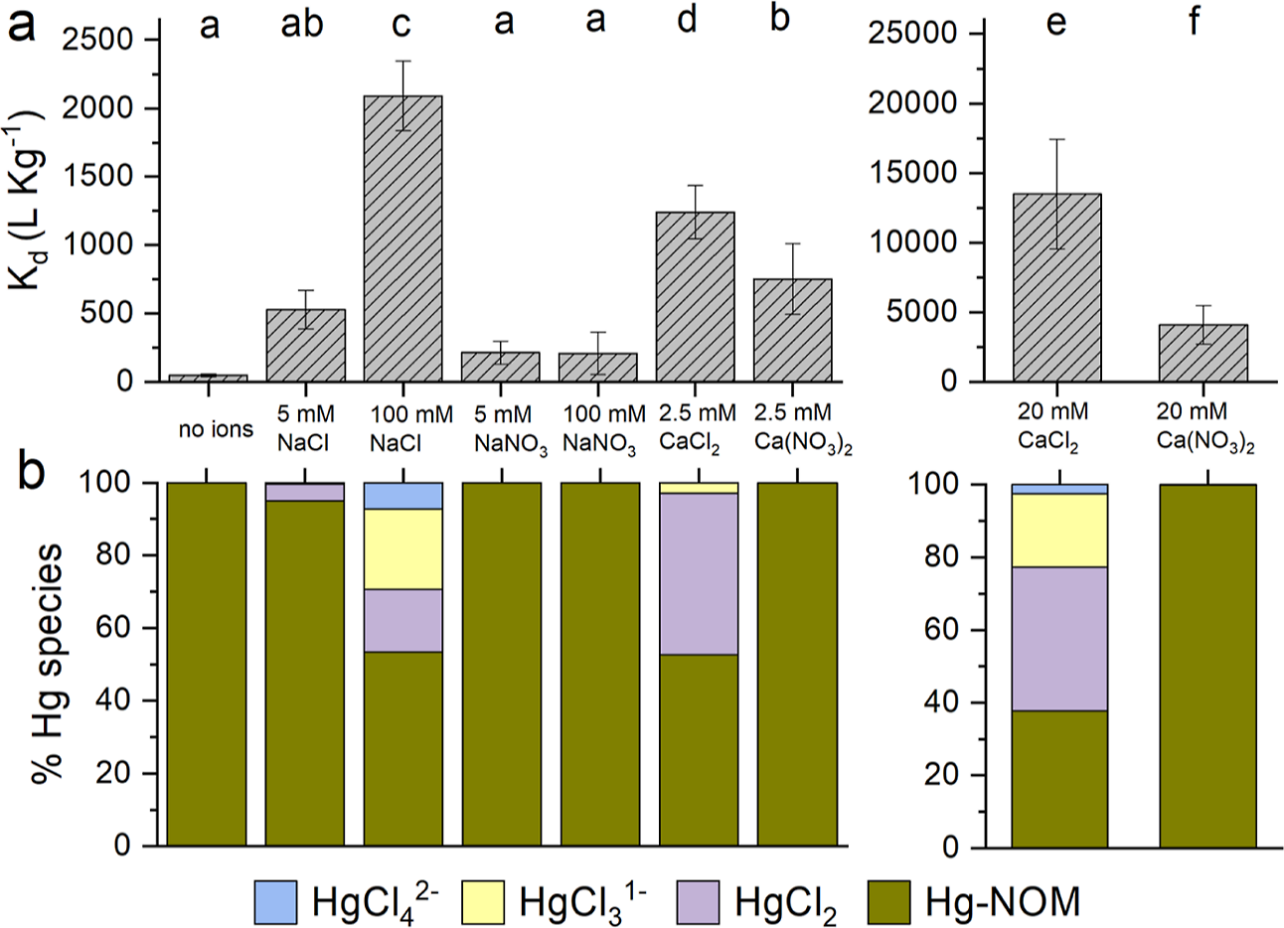
Hg(II) removal by SWP700 in the presence of NOM and different background ions. (a) Sorption coefficients (*K*_d_) of Hg(II) to SWP700 with 20 mg C L^–1^ of NOM (or, 46.2 mg L^–1^ of NOM) and (b) corresponding Hg(II) speciation in the background solution. pH values used in modeling were derived from measurements at the end of the sorption batch experiments, which are shown in Figure S3 of the Supporting Information. Error bars represent standard deviation of at least triplicate measurements. For pairwise comparison of means, the Tukey’s post hoc test was used after running one-way ANOVA (*p* < 0.05).

WHAM VI estimated that with 46.2 mg L^–1^ of NOM, Hg-NOM species become abundant in all solutions (Figure 4b). At Hg/NOM ratios ≤1 μg Hg/mg NOM, Hg(II) is solely associated with strong binding sites in NOM such as reactive S or thiolate groups.^47^ In this study, using a Hg/NOM ratio of 10.8 μg Hg/mg NOM, Hg(II) was also bound to weaker O containing sites in NOM. The moles of reactive *S* in NOM (*S*_NOM, reactive_) were calculated by eq 5. Based on the total *C* and *S* measurements of the lyophilized NOM extract, NOM is 2.31 times the amount of DOC in mg L^–1^ in solution and *S*_total_ is 0.92%. Since Pahokee Peat is an agricultural peat soil from the marshes in the Florida Everglades, we used literature-based values specific to this region for *S*_reduced_ and *S*_reactive_, which were taken to be as 50 and 2%, respectively.^47−49^
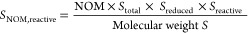
5

The Hg(II)/*S*_NOM, reactive_ molar ratio was calculated to be ∼19:1 in all solutions containing 46.2 mg L^–1^ NOM. Hg(II) was therefore in excess of the reactive S in NOM. Reactive S sites were saturated, and a large fraction of the modeled Hg-NOM complexes consisted of Hg(II) bound to carboxyl and phenolic groups in NOM. Even when considering an upper limit of 30% for *S*_reactive_,^49^ we calculated the Hg(II)/S_NOM, reactive_ molar ratio to be 1.25:1.

We observed that Hg(II) removal increased with increasing NaCl concentration in the presence of NOM, like NOM-free systems (Figure 4a). The speciation of Hg(II) in 100 mM NaCl comprised of Hg(II)-chloro and Hg-NOM complexes. Na^+^ can shield negative charges over SWP700 to allow negatively charged species (HgCl_3_^1-^/HgCl_4_^2-^) to sorb. A slight decrease in negative ζ potential from −32 to −25 mV was observed on increasing the NaCl concentration from 5 mM to 100 mM. However, this is not significant enough to have caused increased Hg(II) removal in the 100 mM NaCl system. Na^+^ can also interact with NOM, and this could increase the hydrophobicity of NOM, causing sorption of NOM and NOM complexed to Hg(II). However, we observed only a minor NOM loss of <5% at the end of the experiments, suggesting that this mechanism was not dominating (Figure S4). Loss of Hg(II) as volatile Hg(0) was also ruled out since experiments were conducted in oxic conditions and contained Cl^–^.^50,51^ Observed XRD indicated an amorphous nature of SWP700, and crystalline Hg_2_Cl_2_ in Cl^–^ rich systems was not evident (Figure S5).

To further investigate the high Hg(II) removal in NaCl NOM systems, we compared the results to NaNO_3_ at similar concentrations. In this case, even with the same amount of Na^+^ present in solution, Hg(II) removal was very low (K_d_ of 200 L kg^–1^), proving that Cl^–^ drove Hg(II) removal in such systems. The presence of high NaCl concentrations allowed Hg(II)-chloro complexes to be formed over time, which were better immobilized than Hg-NOM species. The binding energies of Hg(II) with ligands follow the order of reactive S (log *K* ∼ 21–47) > Cl^–^ as HgCl_2_, HgCl_3_^1–^, or HgCl_4_^2–^ (log *K* ∼ 14–15.54) > O^2–^ as Hg–COO^-^ (log *K* ∼ 7.3–11).^49^ Thus, Hg(II) may have sorbed via a stepwise mechanism of ligand exchange followed by immobilization to the biochar surface: Cl^–^ competed with weakly bound O in NOM for Hg(II) complexation, forming Hg(II)-chloro complexes.^52^ These Hg(II)-chloro complexes having a smaller size than Hg-NOM could probably better access the porous structure of SWP700. Stronger binding reactive S groups within this porous framework could further competitively displace Cl^–^ (eqs 6 and 7).
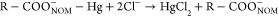
6
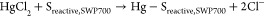
7

The presence of Hg(II) as Hg(II) complexed to S on SWP700 was confirmed by EXAFS spectroscopy, where shell-by-shell fitting of the experimental spectrum yielded 1.8(0.1) S atoms neighboring Hg(II) at 2.32(0.03) Å on SWP700 (Figure S10). This coordination environment is similar to that which has been observed for Hg(II) bound to reactive S or thiol groups.^53,54^ Colocation of Hg(II) and S was further observed in μ-XRF mapping (25 μm beam size), although Cl^–^ and S were seen to be equally colocated with Hg(II) (Figure S7).

The importance of chloro-complexes in this Hg(II) removal mechanism by SWP700 was further confirmed through experiments conducted at a more acidic pH of 5.0 (Figure S13a). Even in the absence of Cl^–^, systems at pH 5.0 showed higher Hg(II) removal than systems at pH 6.9 (*K*_d_ of 494 and 50 L kg^–1^, respectively). This can be attributed to the decreased solubility of NOM and NOM complexed to Hg(II) at lower pH, which allowed more Hg(II) to be removed from solution.^55^ At low pH, O groups tend to be protonated, which decreases the stability of Hg-NOM complexes.^56^ Compared to higher pH scenarios, this enables Cl^–^ to more easily replace those groups as Hg(II) binding partner. As seen from WHAM VI calculations (Figure S13b), a large fraction of Hg(II) existed as HgCl_2_ at pH 5.0 (83%) compared to pH 6.9 (5%). This facilitated more transfer of Hg(II)-chloro complexes to sorption sites within SWP700, at lower pH, which was in good agreement with the proposed stepwise sorption mechanism. Systems at lower pH saw an even larger surge in Hg(II) removal linked to the addition of NaCl: A 5.3-fold increase in *K*_d_ in a 5 mM Cl^–^ system, relative to a system without any Cl^–^, was observed (from 494 to 2600 L kg^–1^), followed by a further 1.7-fold increase in *K*_d_ when increasing Cl^–^ to 100 mM (from 2600 L kg^–1^ to 4430 L kg^–1^).

In addition to Cl^–^ facilitating Hg(II) removal, high Hg(II) removal was also observed in Ca^2+^-rich systems (Figure 4a). WHAM VI determined that 45–60% of Hg(II) in solution existed as Hg(II)-chloro complexes (Figure 4b). Fitting of the EXAFS spectrum to the 20 mM CaCl_2_ sample yielded 2.2(0.2) S neighboring Hg(II) at a distance of 2.4(0.01) Å (Figure S9). Moreover, as seen in in μ-XRF images (25 μm beam size), Hg(II) was correlated with S (Figure S7). Therefore, the mechanisms in eqs 6 and 7 would still dictate Hg(II) removal in the 20 mM CaCl_2_ system. However, this did not fully explain the ∼6.5-fold increase in *K*_d_ compared to the 100 mM NaCl system. Even 2.5 mM CaCl_2_ significantly removed more Hg(II) than 5 mM NaCl.

In suspensions with Ca^2+^, even at very low concentrations, strong NOM aggregation has been reported.^57^ Ca^2+^ can associate multiple NOM molecules electrostatically or through specific binding, increasing the hydrophobicity and aggregation of the supramolecular network of NOM. According to WHAM VI, the charge equivalents per mg of fulvic acids decreased from −5 (in solutions without ions) to −1.5 (in 20 mM CaCl_2_ solutions). The ζ-potential in Ca^2+^-NOM rich systems was in the range of −9 to −15 mV, which was significantly less negative than the range of −26 mV to −36 mV observed in Na^+^-NOM systems (Figure S3). This supports destabilization and increased hydrophobicity of NOM in Ca^2+^-rich systems via cation bridging and charge-shielding effects. Simultaneously, we visually observed the formation of NOM aggregates and measured NOM removal up to 78% from solution at the end of experiments (Figure S4). Due to methodological limitations, it was not possible to distinguish between the truly sorbed and the NOM-coagulated fractions of Hg(II). Aqueous phase recoveries in systems without biochar, though not completely transferrable to systems with biochar, were used to, in part, correct for this (Table S3). Additionally, EDS analysis of the surface of SWP700 showed that Ca^2+^ was present, unlike in pristine SWP700 (Figure S6). In this system, speciation results indicated that 40% of Hg(II) was associated with NOM (Figure 4b). Ca^2+^ therefore removed NOM, including NOM-bound Hg(II) from the aqueous phase. The high Hg(II) removal in CaCl_2_-NOM systems can be explained by a combination of chloro complex formation together with destabilization of NOM/Hg(II)-NOM.

The importance of Ca^2+^ is further emphasized by the high Hg(II) and NOM removal observed in 20 mM Ca(NO_3_)_2_ systems, free of Cl^–^ (Figure 4a). In such systems, the mechanism of chloro complex-mediated Hg(II) removal would no longer be relevant. Although removal with Ca(NO_3_)_2_ was lower than in its CaCl_2_ counterpart, it was still significantly higher than in 100 mM NaCl and 100 mM NaNO_3_. A clear peak of Ca^2+^ on SWP700’s surface was also observed via EDS with 20 mM Ca(NO_3_)_2_ (Figure S6). EXAFS showed a distinct difference in the coordination environment compared to CaCl_2_ and NaCl scenarios (Figure S8). Despite interferences due to beam damage (Figure S12), the peak at ∼2.2 Å could only be fit with a Hg(I)–Hg(I) path (Figure S11). This may be explained by localized reducing environments close to the biochar surface, in line with previous propositions by others.^10,13^ Owing to SWP700’s high pyrolysis temperature, its aromatic π-conjugated system was possibly the primary electron donating center for Hg(II) reduction to Hg(I). This result further underlines the diversity in removal mechanisms for contaminants by biochars under different water chemistry conditions.

### Implications for Industrial Water Treatment

This study elucidated that NOM, ionic composition, and pH are crucial drivers for Hg(II) removal from highly contaminated waters by a wood-based biochar. Hg(II) removal by biochar is generally suppressed in the presence of NOM. Our results show, however, that the ionic composition of contaminated waters has a strong influence on the removal efficiency and may lead to an effective clean-up even at higher NOM concentrations. Specifically, this will occur at high Cl^–^ concentrations where Cl^–^ can effectively displace O-containing moieties in NOM. Hg(II) removal in the presence of Cl^–^ followed a stepwise mechanism: weakly bound oxygen functional groups in NOM were outcompeted by Cl^–^ forming smaller-sized Hg(II)-chloro complexes. Cl^–^ was outcompeted by S groups in biochar which finally immobilized Hg(II). Cations such as Ca^2+^ enhance Hg(II) removal through charge shielding, cation bridging, and aggregation of Hg-NOM complexes.

These processes are dependent on the molar ratios of Hg(II)/NOM, Hg(II)/Cl, and NOM/Cl, as well as the pH. In lesser contaminated waters, low salinity organic matter rich waters, highly alkaline waters (pH > 9), or in waters containing a large fraction of reduced S, the observed effects might differ. This study did not investigate anoxic and sulfidic waste waters, where Hg(II) speciation and removal by biochar will differ.

## Supporting Information Available

Preparation of NOM extract, WHAM VI speciation calculations, aqueous phase Hg(II) recovery in NOM containing background solutions, SWP700 bulk characterization, determination of total Hg using CVAFS, calculation of sorption coefficients (*K*_d_), ζ-potential and pH measurements, removal of NOM, XRD and EDS measurements, generation of μ-XRF plots, EXAFS point selection and data processing, details on EXAFS fitting procedure, and effect of pH on Hg(II) removal in the presence of NOM (PDF)
