# Supplementary material for: Mercury Removal from Contaminated Water by Wood-Based Biochar Depends on Natural Organic Matter and Ionic Composition
Source: Environ Sci Technol. 2022 Aug 4;56(16):11354–62. doi: 10.1021/acs.est.2c01554 (PMC9387100; doi:10.1021/acs.est.2c01554)
Supplement: Supplementary file 1 — es2c01554_si_001.pdf [file es2c01554_si_001.pdf]

Supporting Information

Mercury removal from contaminated water by wood-based biochar depends on natural organic matter and ionic composition

Sampriti Chaudhuri[a,b], Gabriel Sigmund[a], Sharon E. Bone[c], Naresh Kumar[d], Thilo Hofmann[a]*

[a]Department of Environmental Geosciences, Centre for Microbiology and Environmental Systems Science, University of Vienna, Josef-Holaubek-Platz 2, 1090 Vienna, Austria

[b]Doctoral School in Microbiology and Environmental Science, University of Vienna, Josef-Holaubek-Platz 2, 1090 Vienna, Austria

[c]Stanford Synchrotron Radiation Lightsource, SLAC National Accelerator Laboratory, 2575 Sand Hill Rd., Menlo Park, California 94025, United States of America

[d]Soil Chemistry and Chemical Soil Quality Group, Wageningen University, 6708 PB Wageningen, The Netherlands

ORCID:

SC: 0000-0002-8693-8758

GS: 0000-0003-2068-0878

SB: 0000-0002-7521-9627

NK: 0000-0002-8593-5758

TH: 0000-0001-8929-6933

***Corresponding author:** Thilo Hofmann; Phone: +43-1-4277-53320;

Email: thilo.hofmann@univie.ac.at

1. Preparation of NOM extract

The bulk material (IHSS-2BS103P) was ground and homogenized at 30 Hz for 20 seconds on a Retsch Mixer Mill MM 400 (Retsch GmbH, Germany). 50 g L^{-1} of ground Pahokee Peat in 0.05 M sodium hydroxide (NaOH) was sonicated at 35 kHz (Sonorex Super RK 106, Bandelin Electronics GmbH, Germany) in 40 mL pre-cleaned (rinsed with isopropanol and milli-Q water) polypropylene tubes for 1 h. The solutions with fine solids were shaken for 24 h on a horizontal shaker at 125 rpm. After centrifuging at 17,000 g for 2 h using a Sorvall LYNX 6000 superspeed centrifuge (Thermo Fischer Scientific, USA), all solids were effectively separated from the aqueous and suspended phases. The solutions were passed through 0.22 µm Millipore filters using a vacuum filtration unit and dialyzed with a regenerated cellulose membrane (Spectrum Laboratories, USA), having a molecular weight cut off <1 kDa. After dialyzing against milli-Q water for ~7 days by repeatedly changing the spent water twice a day (till the conductivity was < 1 µS cm^{-1}), the DOC of the extract was measured on a TOC analyzer.

150 mL of this DOC extract was lyophilized over 72 hours (Freeze dryer Alpha 1-4 LSC, Martin Christ Gefriertrocknungsanlagen GmbH, Germany), and analyzed on the elemental analyzer (Elementar VarioMacro, Elementar Analysensysteme GmbH, Germany) for total C and S. Using a measured C composition of 43.2% (w/w) basis, this DOC based concentration of 1200 mg L^{-1} is calculated to be 2778 mg L^{-1} of NOM. We used DOC concentrations as a measure of NOM concentration throughout the study.

2. WHAM VI speciation calculations

A detailed description of the Humic Ion-Binding Model VI is provided in Tipping 1998.[1] Briefly, WHAM is a discrete site/electrostatic model for humic-ion binding, containing strong metal affinity type A (mainly carboxylic, but can be thiolic as well) and weak metal affinity type B sites (phenolic) in humic acids (HA) and fulvic acids (FA). The binding of metals at sites A and B are dependent

on the intrinsic binding constant (K_{MA} and K_{MB}), and a 3rd parameter ΔLK_2 which describes the interactions of the metal with the strong "softer" ligands (S in the case of Hg). Numerous literature shows that the value of ΔLK_2 will have a strong influence on the Hg(II) complexation ability to humic substances, as detailed in Tipping 2007.[2] Therefore, to not overestimate Hg(II)-NOM binding, we modified our modelling parameters. Like the best fit scenario in the works of Richard et al., 2016,[3] we used log K_{MA} values of 3.1 and 3.3 for FA and HA respectively and set the ΔLK_2 to 1.7. ΔLK_2 was reduced from the default value of 5.1, to ensure that it did not account for thiolic sites. The thiolic fraction (RS^-) was input separately, independent of the HA and FA phases. Equilibrium constants used are provided in Table S1.

Table S1: Used equilibrium constants for Hg(II) complexes

Name	Reaction	Equilibrium constants (log K)	
$Hg(SR)_2$	$2RS^- + Hg^{2+} = Hg(SR)_2$	42#	
$Hg(SR)^+$	$RS^- + Hg^{2+} = Hg(SR)^+$	20#	
$HgCl^+$	$Hg^{2+} + Cl^- = HgCl^+$	7.21	
$HgCl_2$	$Hg^{2+} + 2Cl^- = HgCl_2$	13.98	
$HgCl_3^{1-}$	$Hg^{2+} + 3Cl^- = HgCl_3^{1-}$	15.06	
$HgCl_4^{2-}$	$Hg^{2+} + 4Cl^- = HgCl_4^{2-}$	15.42	
$HgOH^+$	$Hg^{2+} + OH^- = HgOH^+$	10.6	
$Hg(OH)_2$	$Hg^{2+} + 2OH^- = Hg(OH)_2$	21.83	
$Hg(OH)_3^-$	$Hg^{2+} + 3OH^- = Hg(OH)_3^-$	20.9	
RSH	$RS^- + H^+$	-10#	
Name	Reaction	log K_{MA}	ΔLK_2
$Hg\text{-}R^+_{\sim fulvic\ acid}$	$Hg^{2+} + R\sim = HgR^+\sim$	3.1	1.7
$Hg\text{-}R^+_{\sim humic\ acid}$	$Hg^{2+} + R\sim = HgR^+\sim$	3.3	1.7

Ref: Skyllberg 2008, Richard et al., 2016[3,4]

Since we did not fractionate our NOM, it contained both the hydrophobic and hydrophilic fractions. We assumed that the distribution of available Hg(II) complexing sites was concentrated rather in the hydrophobic acid fraction, with negligible contribution from the hydrophilic fraction.[3,5,6] Pahokee Peat is a peat soil coming from the freshwater marshes of the Florida Everglades,[7] so

most of our assumptions (Table S2) are based on previous studies conducted using Florida Everglades organic matter.

Table S2: Parameters used for calculating FA, HA and thiol groups in WHAM VI modeling

Parameter	*Value used as input*	*Reference*
[NOM] in mg L^{-1}	2.31 * [DOC]	Measured (this study)
Hydrophobic NOM in %	49% of [NOM]	Poulin et al., 2017 [8]
FA and HA of hydrophobic NOM in %	96% and 4% of hydrophobic [NOM]	Hoch et al., 2000 [9]
Total S in NOM in %	0.92% of [NOM]	Measured (this study)
Reduced S in NOM in %	50% of total S	Khwaja et al., 2006 and Dong et al., 2010 [10,11]
Reactive S available for Hg(II) binding in %	2% of Reduced S #	Haitzer et al., [12]

We also simulated the Hg(II) speciation at 30% of reduced S as reactive S, as an upper limit[11] (Figure S1). The fraction of NOM bound Hg(II) only changed in 3 scenarios (100 mM NaCl, 2.5 mM $CaCl_2$, 20 mM $CaCl_2$), but this will not change the interpretation of our data, since Hg(II)-chloro complexes can still be formed according to the model.

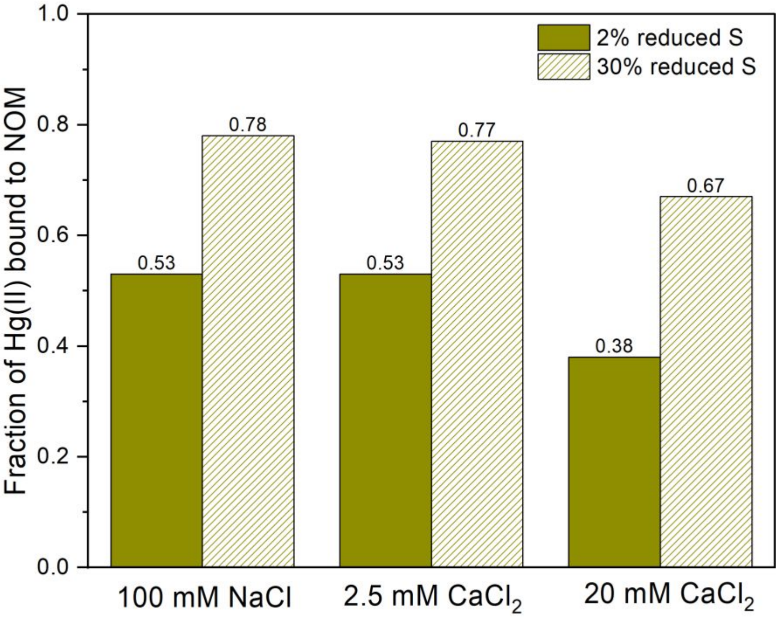

Figure S1. Fraction of Hg(II) bound to NOM when % of reactive S was varied

3. Aqueous phase Hg(II) recovery in NOM containing background solutions

Table S3: Aqueous phase % recovery of Hg(II) in background solutions containing NOM

Solution	*% Hg recovered unfiltered*	*% Hg recovered filtered (0.45 μm)*
46.2 mg/L NOM + 5 mM NaCl	101 ± 4%	99 ± 6%
46.2 mg/L NOM + 100 mM NaCl	107± 3%	108 ± 5%
46.2 mg/L NOM + 5 mM $NaNO_3$	98 ± 1%	94 ± 7%
46.2 mg/L NOM + 100 mM $NaNO_3$	103 ± 4%	97 ± 1%
46.2 mg/L NOM + 2.5 mM $CaCl_2$	93 ± 3%	92 ± 3%
46.2 mg/L NOM + 20 mM $CaCl_2$	77 ± 1% [#]	78 ± 3% [#]
46.2 mg/L NOM + 2.5 mM $Ca(NO_3)_2$	102 ± 2%	101 ± 2%
46.2 mg/L NOM + 20 mM $Ca(NO_3)_2$	35 ± 4% [#]	32 ± 5% [#]

[#] Centrifugation caused Ca^{2+} aggregated NOM/Hg-NOM to settle, reducing the amount recovered in the supernatant. In 20 mM $CaCl_2$ system, since Hg(II) is also complexed to Cl^-, the aqueous phase recovery is higher than in the 20 mM $Ca(NO_3)_2$ system. In the 20 mM $Ca(NO_3)_2$ system, Hg(II) would be complexed entirely to Ca^{2+} aggregated NOM, which settles partially after centrifugation.

4. SWP700 bulk characterization

SWP700 was pyrolyzed from 5:95 pine:spruce mixed wood at 700°C in a pilot-scale rotary kiln at the UK Biochar Research Centre (UKBRC, Edinburg, UK). Production details and system operation have been specified elsewhere.[13]

The microstructure and morphology of SWP700 was examined using an Inspect™ S50 scanning electron microscope (SEM) equipped with an Everhart-Thornley detector (FEI, USA), operating at 15 kV accelerating voltage (magnified between 100x to 1500x) at a working distance of 10.1±0.2 mm. The specific surface area of SWP700 was obtained following protocols of Sigmund et al.[14] from N_2 physisorption isotherms after overnight degassing at 105°C using a Quantachrome Nova 2000 analyzer (Quantachrome Instruments, USA). Ash content was determined from the weight loss of sample after heating at 750°C for 6 h.[15] Determination of total C, H, N, S and of

SWP700 was done using an elemental analyzer (Elementar VarioMacro, Elementar Analysensysteme GmbH, Germany). O content was calculated by difference: O% = 100 – (C + H + N + S + ash). Total metal content was determined by digesting duplicates of 20 mg of SWP700 on a hot plate through stepwise addition of nitric acid (65% suprapure HNO_3, Merck), hydrochloric acid (30% suprapure HCl, Roth), hydrofluoric acid (40% suprapure HF, Merck), and hydrogen peroxide (suprapure H_2O_2, Merck) as detailed in a previous study.[16] Results are shown in Figure S2 and Table S4.

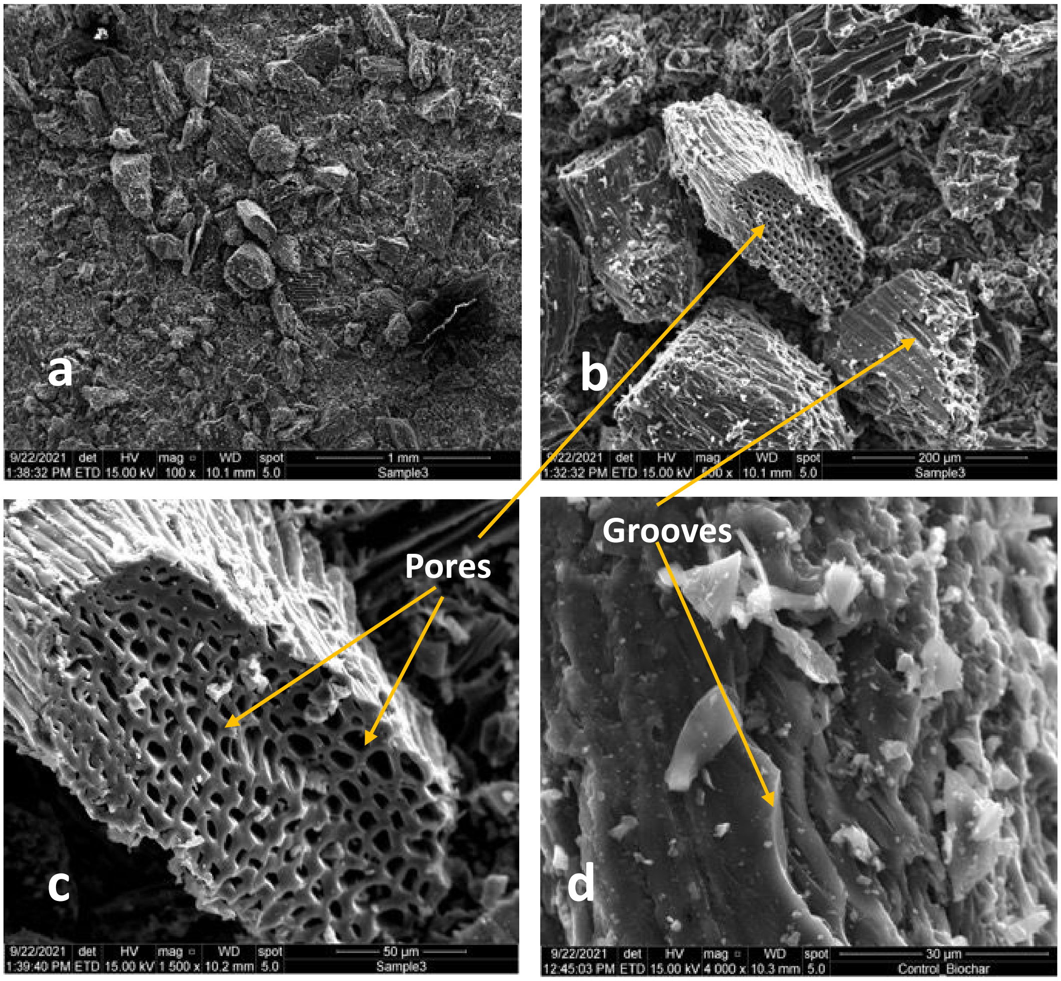

Figure S2. Scanning electron micrographs (SEM) images of SWP700. Magnification of (a) 100, (b) 500, (c) 1500 and (d) 4000

Table S4. Results of Characterization of SWP700

Specific Surface Area (m^2/g)	184.43	
C%	89.95	
H%	1.63	
N%	0.25	
S%	0.18	
Ash%	1.89	
O%	6.09	
H:C (molar ratio)	0.22	
O:C (molar ratio)	0.05	
Metal content (mg kg^{-1})	Al	279
	Ca	4182
	Fe	183
	K	1811
	Mg	930
	Mn	238
	Na	200

5. Determination of total Hg using CVAFS

The instrument detection limit was 10 ng L^{-1}. Hg(II) calibration and quality control standards were prepared from a standard NIST 3133 solution of mercuric nitrate ($Hg(NO_3)_2$, NIST, USA). 0.2N bromine monochloride (BrCl) solution was prepared according to protocols of Bloom et al.[17] All collected filtered samples were reacted with 1% (v/v) 0.2N BrCl for at least 24 h to oxidize all Hg in solution to Hg^{2+}. All necessary dilutions were made using 1% (v/v) BrCl. Prior to analysis, a small aliquot (20μL) of reagent grade hydroxylammonium chloride (NH_2OH:HCl, Merck) was added to 10 mL of diluted samples to destroy excess halogens. Following reduction using 2% reagent grade tin(II) chloride ($SnCl_2$) (w/v) in 2% HCl, samples were purged with ultrahigh purity N_2. The

emerging Hg(0) was adsorbed onto a gold trap, and subsequently thermally desorbed for detection and quantification on the spectrophotometer.

6. Calculation of sorption coefficients (K_d)

The amount of Hg(II) immobilized by SWP700 and the equilibrium sorption coefficients were calculated based on loss from the aqueous phase, using the following equations:

$$q_e = \frac{V(C_0 - C_e)}{W} \quad \text{(eq 1)}$$

$$K_d = \frac{q_e}{C_e} \quad \text{(eq 2)}$$

where q_e (mg kg^{-1}) is the mass of Hg(II) sorbed per unit mass of SWP700 at equilibrium; C_0 and C_e (mg L^{-1}) are the initial and equilibrium Hg(II) concentrations in the aqueous phase respectively; W(kg) is the mass of SWP700; V(L) is the volume of the aqueous phase and K_d (L kg^{-1}) is the equilibrium sorption coefficient.

7. ζ-potential and pH measurements

Tubes were agitated manually by hand and larger particles were allowed to settle for 90 seconds. Immediately after, the sample was transferred to an Omega cuvette and the ζ-potential was measured on a Litesizer™ 500 (Anton Paar, Austria) via electrophoretic light scattering (ELS) at room temperature. An equilibration time of 1 minute, adjusted maximal voltage ranging between 72 V to 200 V, and 100 runs were used per measurement. ζ-potential was calculated from the electrophoretic mobility using the Smoluchowski approximation. All samples were measured in duplicates.

The pH was measured using a SenTix 81 pH electrode (Xylem Analytics, Germany) calibrated using pH buffers 4 and 7 (Merck, Germany). Results are shown in Figure S2.

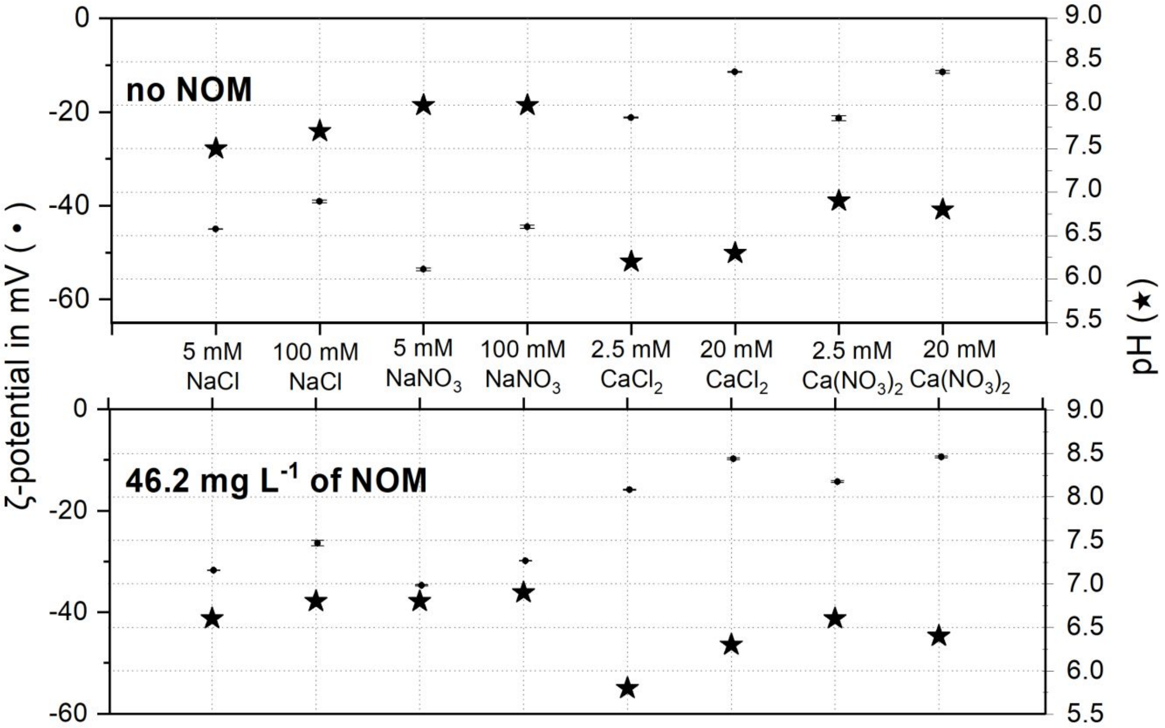

Figure S3. ζ-potential measurements (•) with the corresponding pH (★) in NOM-free and NOM containing systems. Standard deviation of duplicate ζ-potential measurements represented by error bars.

8. Removal of NOM

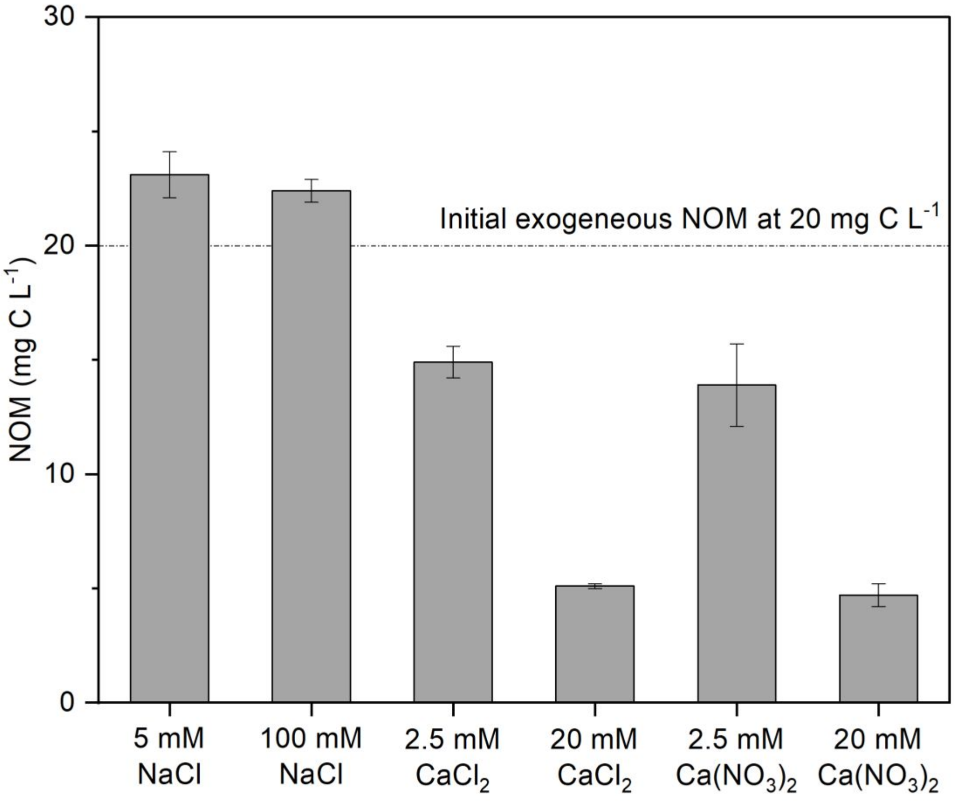

Figure S4. NOM levels at the end of experiments. 2-3 mg L^{-1} of DOC is leached out from SWP700 inherently. Standard deviation of duplicate samples is represented by error bars. The observed high removal of Hg(II) and NOM with 20 mM $CaCl_2$ and $Ca(NO_3)_2$ should be treated with caution, since only a fraction of destabilized NOM could have sorbed to SWP700. In the scope of our experiments, it was not possible to discriminate between the dissolved Hg-NOM and particulate Hg-NOM forms such as flocs.

9. XRD and EDS measurements

X-ray diffraction patterns of vacuum dried samples (reacted and un-reacted SWP700) were collected using a Rigaku MiniFlex 600 diffractometer equipped with a monochromator and a Cu Kα radiation (λ = 1.54 Å) X-ray tube operating at 20 kV and 15 mA. Ground to powder samples were analyzed for 2θ range of 10 to 90° using a zero-background silicon base holder and International Center for Diffraction Database (ICDD) was used to determine mineral phases. Energy dispersive X-ray spectroscopy (EDS) of the samples was carried out using the Inspect S50 (FEI, USA), in the EDS analysis mode at an accelerating voltage of 15 kV. At a magnification of 100x and working distance of 10.1 mm, 3 spots were selected at random to check for the presence of Ca, Na and Cl.

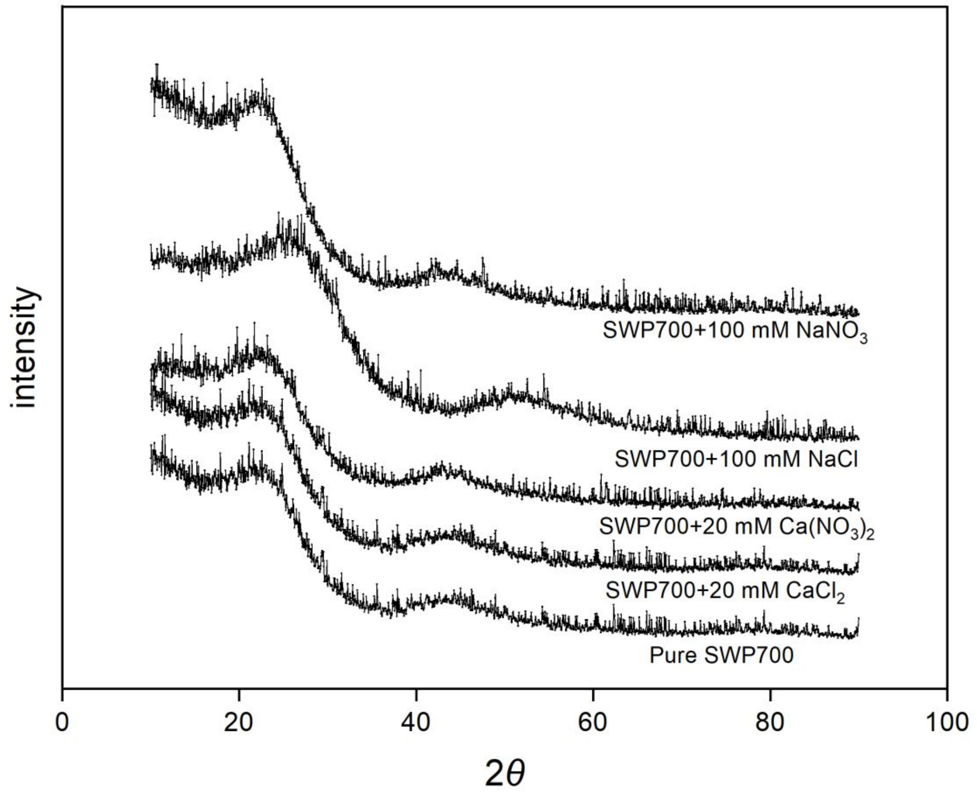

Figure S5. X-Ray Diffractograms (XRD) of un-reacted SWP700 and SWP700 after sorption experiments in systems with 46.2 mg L^{-1} NOM and different background ions.

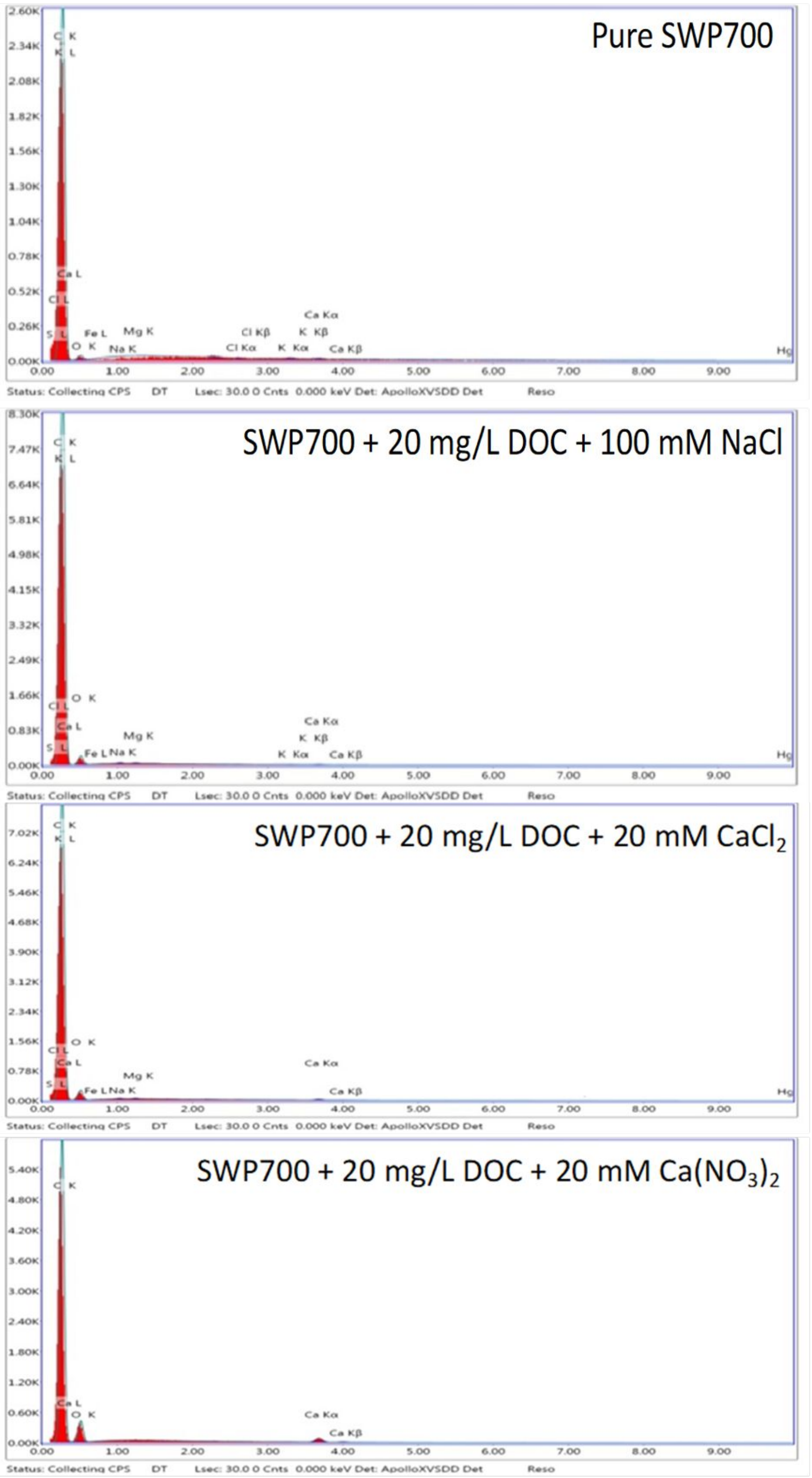

Figure S6. EDS scans of pure SWP700 and SWP700 after sorption experiments in systems with 20 mg L^{-1} of DOC (or 46.2 mg L^{-1} NOM) and different background ions

10. Generation of μ-XRF plots, EXAFS point selection, and data processing

The monochromator was calibrated by setting the first derivative of a Au foil K-edge absorption spectrum to 11919.0 eV. Fluorescence was monitored using a four-element Vortex detector. Specifically, images obtained by μ-XRF mapping were analyzed using Sam's microprobe analysis toolkit (SMAK).[18] The S, Cl, and Hg fluorescence intensities for each pixel in the two-dimensional image were obtained by fitting the energy-dispersed spectrum recorded by the Vortex detector using a set of Gaussian peaks via the pyMCA module in SMAK. For each sample, a plot of Hg versus Cl and Hg versus S intensity was generated to assess whether chemically distinct areas of varying Hg-S or Hg-Cl associations occurred (Figure S7). None were observed, thus for EXAFS spectrum generation, we selected a 25- μm spot with relatively high Hg intensities to ensure adequate signal:noise.

Replicate EXAFS scans were compared to assess whether beam damage occurred. Beam damage was not observed in the samples in contact with 100 mM NaCl and 20 mM $CaCl_2$, but was observed in the one with 20 mM $Ca(NO_3)_2$, as shown in Figure S11. Replicate scans were averaged and then background subtracted and normalized by fitting a line to the pre-edge region (12139.9 eV – 12244.9 eV) and a third-order polynomial to the post-edge region (12439.9 eV – 12761.8 eV). The EXAFS were extracted using a spline from k = 0 – 12.2 $Å^{-1}$. The R_{bkg} value, which determines the lower limit in R-space to which the background function is fit, was set to be 1.16 for the $Ca(NO_3)_2$ sample. The theoretical scattering paths that were used to produce the models of the FT-EXAFS spectra were calculated in FEFF 6 using atomic coordinated for 5-Å diameter α-HgS and Hg_2Cl_2.[19,20]

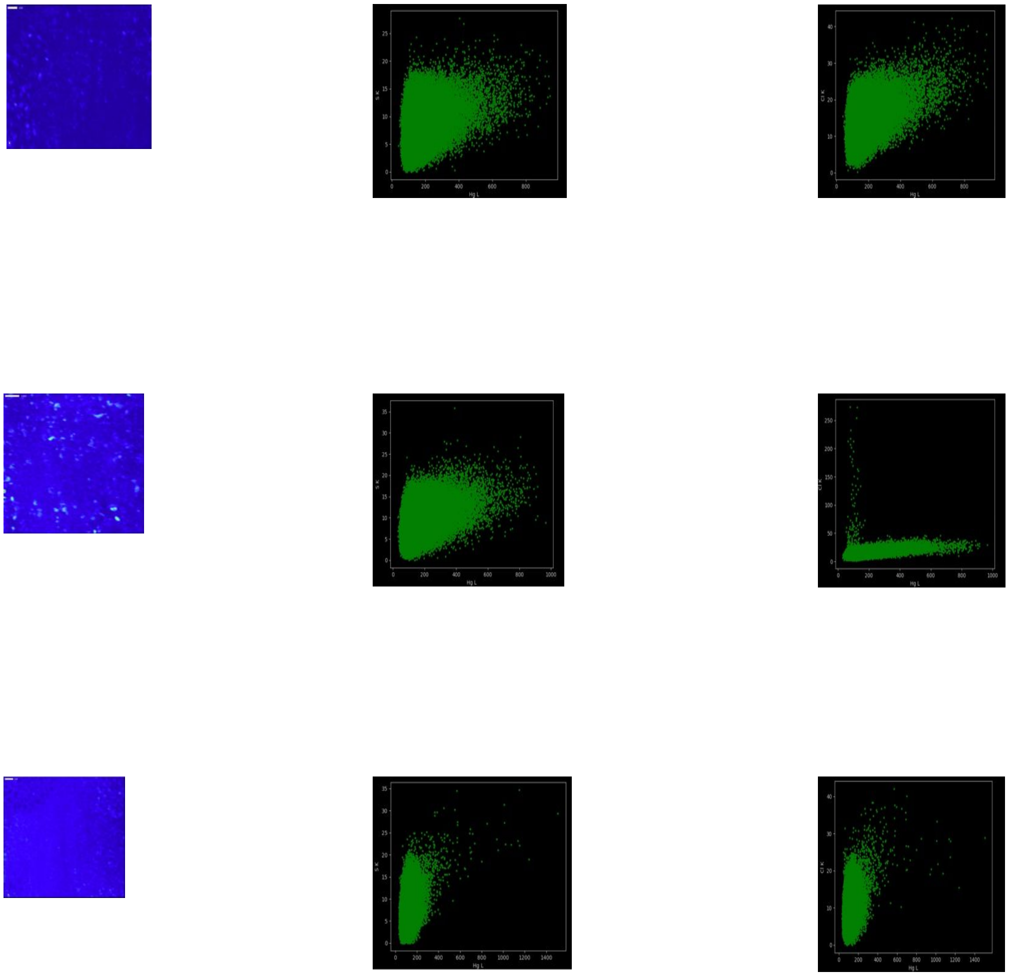

Figure S7: μ-XRF maps of Hg. Heat maps of Hg in SWP700 with 100 mM NaCl, 20 mM $CaCl_2$, and 20 mM $Ca(NO_3)_2$ from top to bottom (red denotes high intensity, blue denotes low intensity). Maps were collected with 25 μm resolution. The position where the μ-EXAFS scan was collected is shown by the small white circle (see arrow). Correlation plots of the Hg intensity versus the S or Cl intensity for each pixel within the images are also shown. Hg is weakly correlated with both S and Cl in all samples.

11. Details on EXAFS fitting procedure

Shell-by-shell fits were performed to determine the speciation of Hg in three samples. We fit a separate energy shift (ΔE; eV; used to align the theoretical scattering paths with the experimental data), for Hg-S and Hg-Hg scattering paths. For each scattering path we fit the bond distance (R; Å), the Debye-Waller factor, a measure of structural and thermal disorder (σ^2; Å^2), as well as the amplitude. The amplitude was equal to the product of the number of coordinating atoms (N) and the amplitude reduction factor (S_0^2). The S_0^2 value was set to 0.8.[21]

Fits to Hg-loaded sample in 20 mM $CaCl_2$ system revealed Hg coordinated to ~two S atoms at 2.40(0.01) Å (Figure S9). The bond distance is slightly longer than what is predicted for a two-coordinate Hg-thiol complex (~2.34 Å),[22] which might indicate the presence of Hg-S species with a higher coordination numbers of 3 or 4, which exhibit longer Hg-S bond distance (~2.45 and 2.55 Å, respectively).[23]

Like the previous sample, the Hg-loaded sample in 100 mM NaCl system was fit with Hg coordinated to ~2 S atoms at a bond distance consistent with linear coordination, 2.32(0.03) Å (Figure S10). However, we obtained a poor fit (R-factor = 0.11; data not shown) unless we also included skew and kurtosis as fitting parameters. Skew (σ^3; Å^3) and kurtosis (σ^4; Å^4) are the third and fourth moments of a Gaussian distribution and account for anharmonicity in Hg bonding.[24] This anharmonicity could arise if Hg exhibits a distorted coordination environment, yielding peaks in the Fourier transformed (FT)-EXAFS spectra that cannot be resolved within the k-range measured.

We fit the Hg-loaded sample for 20 mM $Ca(NO_3)_2$ system, despite the presence of beam damage, which impacted the Hg coordination environment. Although we cannot assess what the undamaged Hg speciation would be, the EXAFS oscillations of the first scan exhibited the same frequency as the average of all 40 scans collected, indicating that the dominant frequency apparent in the initial EXAFS spectrum persisted after beam damage and could reveal pertinent

information about Hg speciation in the sample (Figure S12). This sample exhibited a distinct coordination environment relative to the other two (Figure S8). The peak at ~2.2 Å in the FT-EXAFS spectrum could only be fit with a Hg(I)-Hg(I) scattering path taken from Hg_2Cl_2 (Figure S11). We also fit a smaller contribution from Hg coordinated to S, like what was observed for the other two samples. However, both the Hg-S and Hg-Hg bond distances were significantly contracted (~0.2 Å) relative to the distances in the model compounds used to obtain the FEFF scattering paths and the ΔE value was greater than ± 10 eV. Nonetheless, the ~2.2-Å peak in the FT-EXAFS spectrum could not be fit with Hg(II)-S, Hg(II)-Cl, or Hg(II)-O scattering paths.

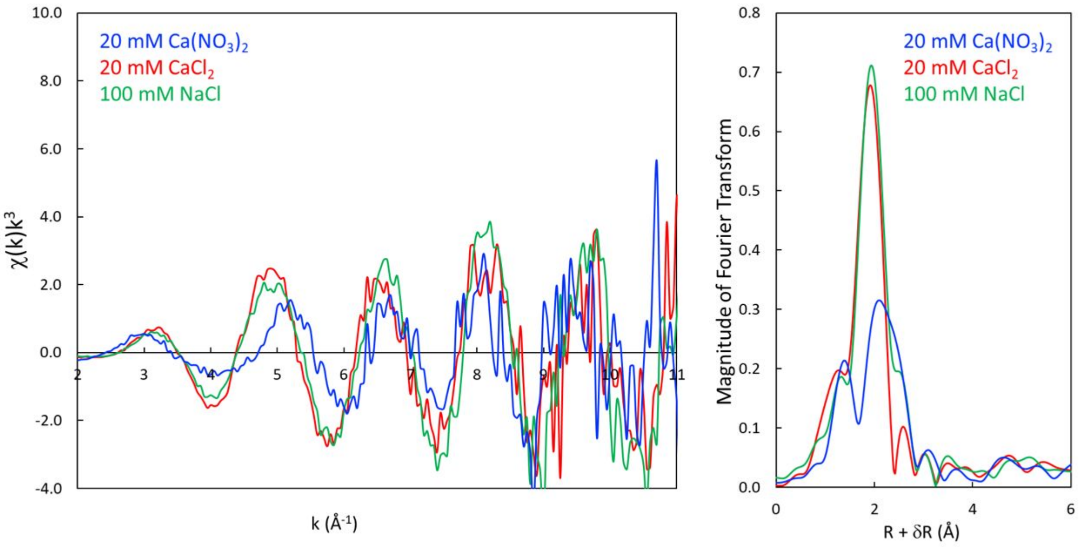

Figure S8. EXAFS spectra (left) and Fourier transformed-EXAFS spectra (right) from samples loaded with Hg(II) after sorption experiments.

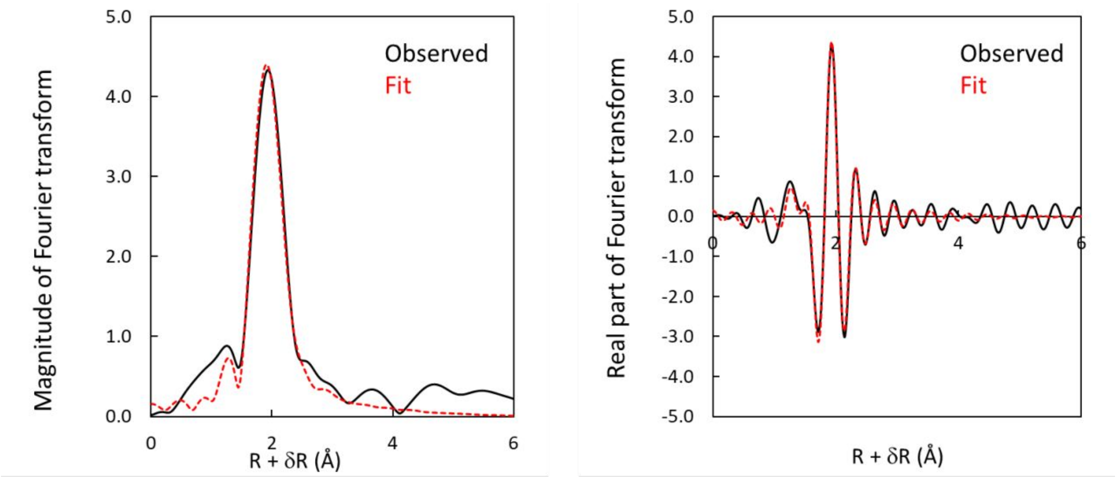

Scattering path	S02	CN	error on CN	R (Å)	error on R	σ^2 (Å^2)	error on σ^2	ΔE	error on ΔE	R-value	R range	k range
Hg-S	0.8	2.2	0.2	2.399	0.009	0.004	0.001	-1.26	1.07	0.025	1 to 3.5	2 to 10.3

Figure S9. Fourier transformed EXAFS spectra (solid lines), fits (dotted lines) and fitting parameters (bottom table) of Hg-loaded SWP700 sample originating from the 20 mM $CaCl_2$ system.

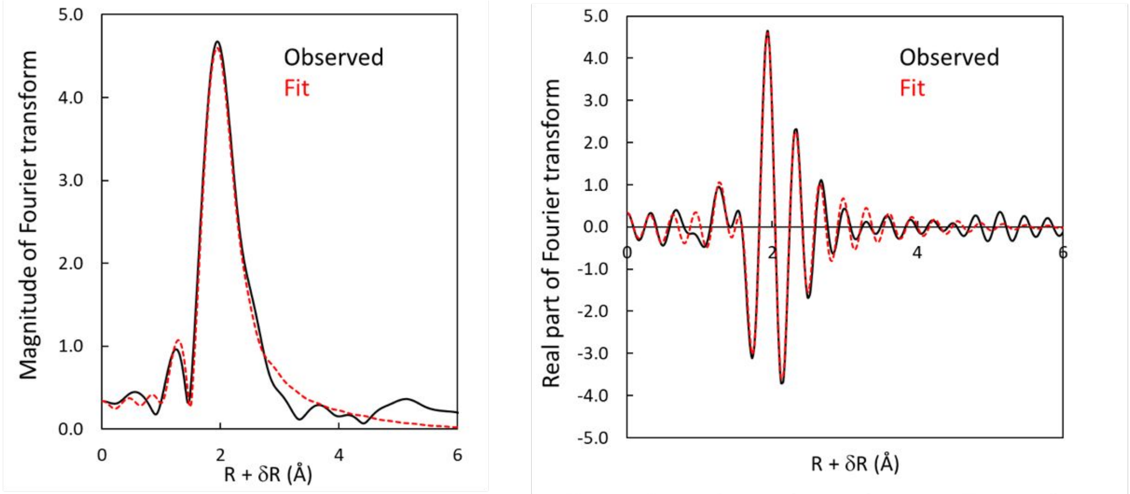

Scattering path	S02	CN	error on CN	R (Å)	error on R	σ^2 (Å^2)*	error on σ^2	ΔE	error on ΔE	σ^3 (Å^3)	error on σ^3	σ^4 (Å^4)	error on σ^4	R-value	R range	k range
Hg-S	0.8	1.8	0.1	2.315	0.031	0.0039		-5.93	2.23	-0.0013	0.0005	0.00012	0.00003	0.021	1 to 3.5	2 to 10.3

Figure S10. Fourier transformed EXAFS spectra (solid lines), fits (dotted lines) and fitting parameters (bottom table) of Hg-loaded SWP700 sample originating from the 100 mM NaCl system.

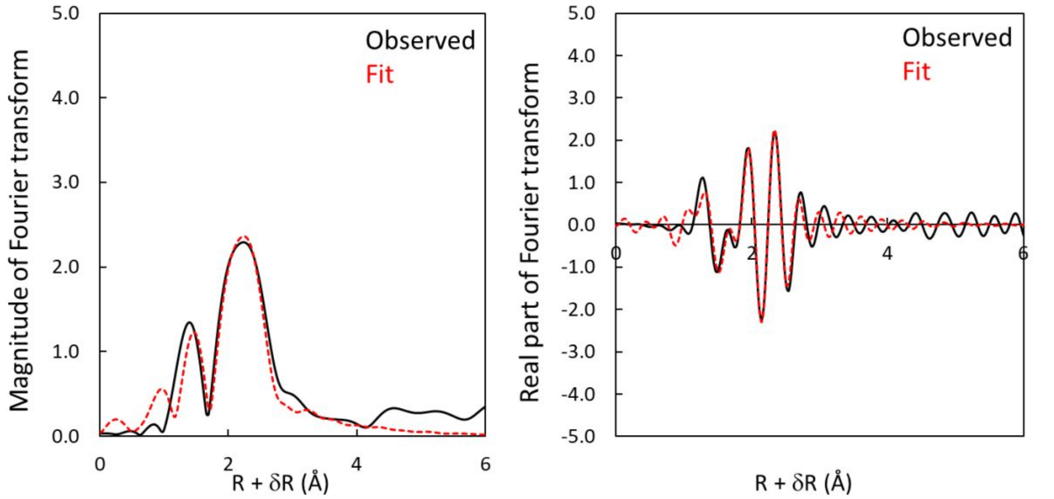

Scattering path	S02	CN	error on CN	R (Å)	error on R	σ^2 (Å^2)*	error on σ^2	ΔE	error on ΔE	R-value	R range	k range
Hg-S	0.8	3.5	1.1	2.206	0.022	0.014	0.004	-13.50	2.46	0.051	1.16 to 3.5	2 to 10.3
Hg-Hg	0.8	3.8	1.8	2.299	0.015	0.008	0.003	-11.39	2.86			

Figure S11. Fourier transformed EXAFS spectra (solid lines), fits (dotted lines) and fitting parameters (bottom table) of Hg-loaded SWP700 sample originating from the 20 mM $Ca(NO_3)_2$ system.

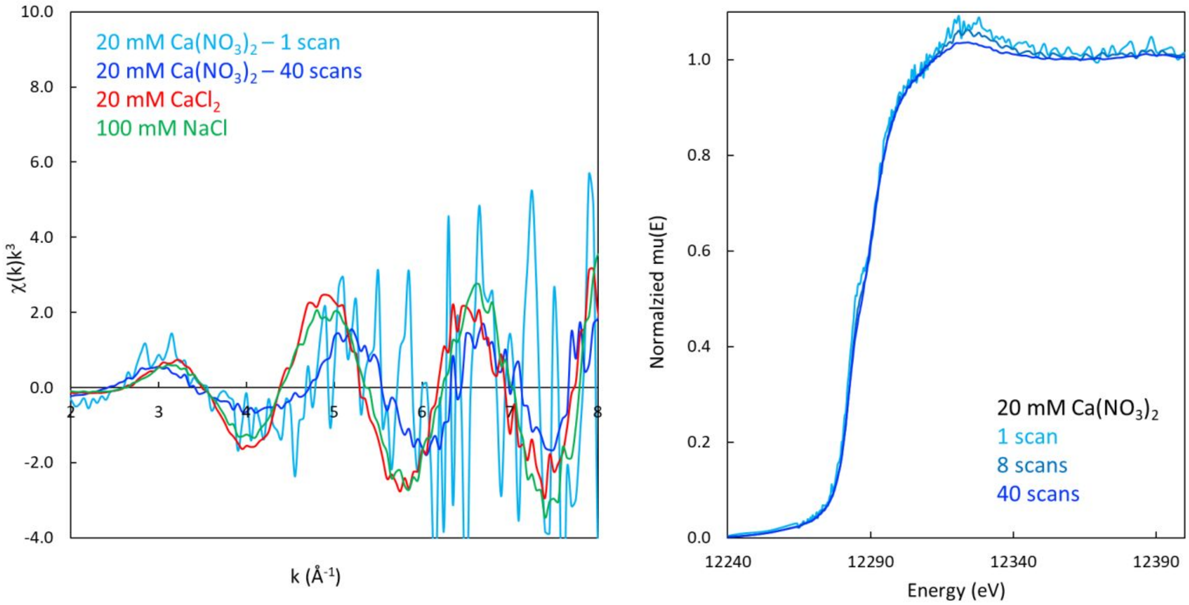

Figure S12. Beam damage in the Hg-loaded sample originating from the 20 mM $Ca(NO_3)_2$ system. Beam damage was observed in the sample containing 20 mM $Ca(NO_3)_2$, which can be seen by comparing the first XANES scan to the average of the first 8 XANES scans and the full set of 40 XANES scans that were collected on this sample (right). The spectral features change more greatly between scans 1 and 8 than between scans 8 and 40, suggesting that damage is initially rapid, and then slows down. Nonetheless, the EXAFS of the first scan exhibit Hg oscillations of the same frequency as the EXAFS resulting from the 40-scan average, within noise (left). Although we cannot fit the Hg speciation, unimpacted by beam damage, regardless of beam damage, this sample clearly exhibits a Hg bonding environment that is distinct from the other two.

12. Effect of pH on Hg(II) removal in the presence of NOM

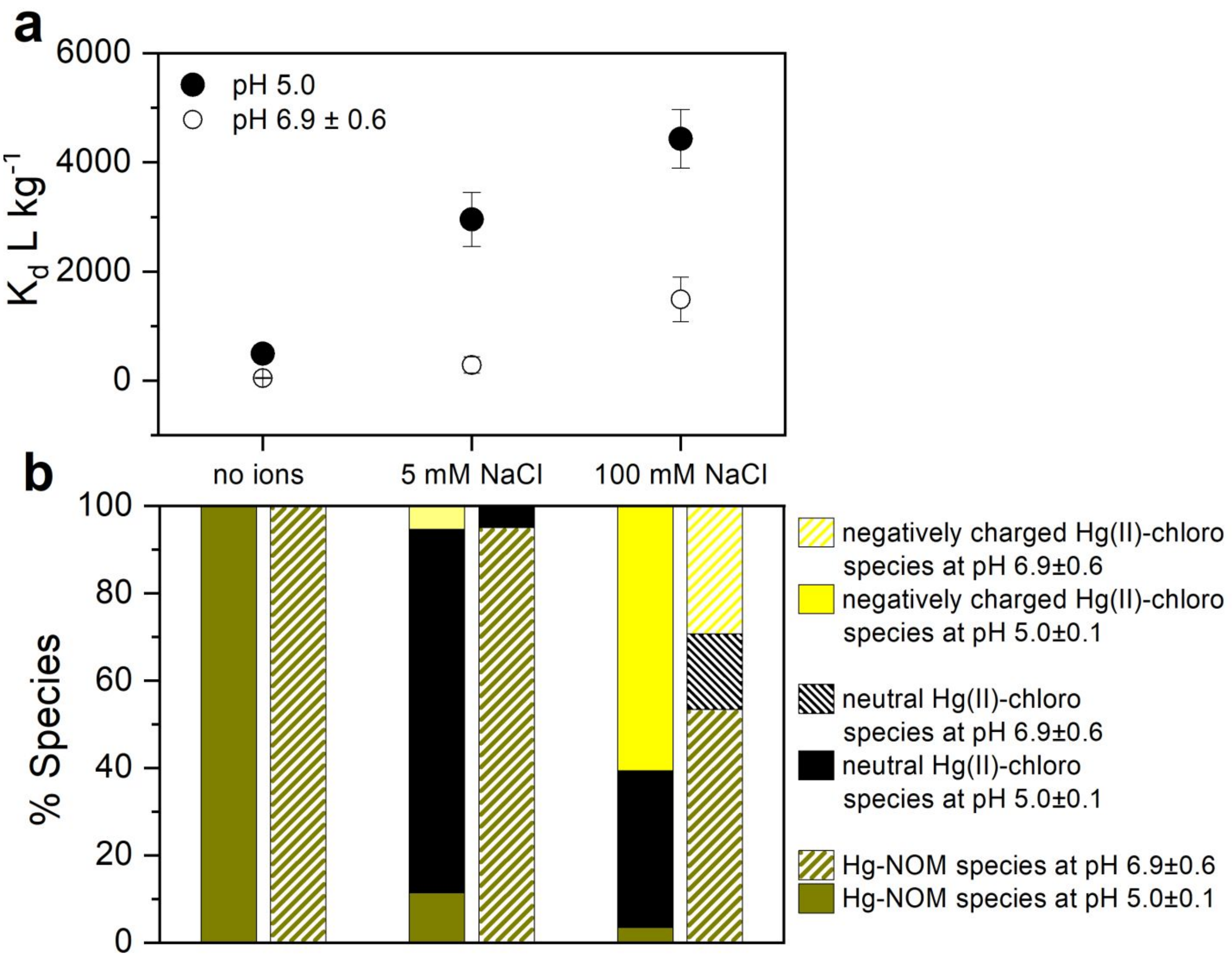

Figure S13. Effect of pH on Hg(II) removal in systems with 46.2 mg L^{-1} of NOM under different concentrations of NaCl. **(a):** K_d at buffered pH 5 (●) vs unbuffered pH 6.9 (○); **(b):** Corresponding Hg(II) speciation at pH 5.0 (filled bars) vs pH 6.9 (unfilled bars).

References

(1) Tipping, E. Humic Ion-Binding Model VI: An Improved Description of the Interactions of Protons and Metal Ions with Humic Substances. *Aquat. Geochemistry* **1998**, *4* (1), 3–47. https://doi.org/10.1023/A:1009627214459.

(2) Tipping, E. Modelling the Interactions of Hg(II) and Methylmercury with Humic Substances Using WHAM/Model VI. *Appl. Geochemistry* **2007**, *22* (8 SPEC. ISS.), 1624–1635. https://doi.org/10.1016/j.apgeochem.2007.03.021.

(3) Richard, J. H.; Bischoff, C.; Biester, H. Comparing Modeled and Measured Mercury Speciation in Contaminated Groundwater: Importance of Dissolved Organic Matter Composition. *Environ. Sci. Technol.* **2016**, *50* (14), 7508–7516. https://doi.org/10.1021/acs.est.6b00500.

(4) Skyllberg, U. Competition among Thiols and Inorganic Sulfides and Polysulfides for Hg and MeHg in Wetland Soils and Sediments under Suboxic Conditions: Illumination of Controversies and Implications for MeHg Net Production. *J. Geophys. Res. Biogeosciences* **2008**, *113* (G2), n/a-n/a. https://doi.org/10.1029/2008jg000745.

(5) Ravichandran, M.; Aiken, G. R.; Ryan, J. N.; Reddy, M. M. Inhibition of Precipitation and Aggregation of Metacinnabar (Mercuric Sulfide) by Dissolved Organic Matter Isolated from the Florida Everglades. *Environ. Sci. Technol.* **1999**, *33* (9), 1418–1423. https://doi.org/10.1021/es9811187.

(6) Dittman, J. A.; Shanley, J. B.; Driscoll, C. T.; Aiken, G. R.; Chalmers, A. T.; Towse, J. E. Ultraviolet Absorbance as a Proxy for Total Dissolved Mercury in Streams. *Environ. Pollut.* **2009**, *157* (6), 1953–1956. https://doi.org/10.1016/j.envpol.2009.01.031.

(7) International Humic Substances Society. Source Materials for IHSS Samples https://humic-substances.org/source-materials-for-ihss-samples/.

(8) Poulin, B. A.; Ryan, J. N.; Nagy, K. L.; Stubbins, A.; Dittmar, T.; Orem, W.; Krabbenhoft, D. P.; Aiken, G. R. Spatial Dependence of Reduced Sulfur in Everglades Dissolved Organic Matter Controlled by Sulfate Enrichment. *Environ. Sci. Technol.* **2017**, *51* (7), 3630–3639. https://doi.org/10.1021/acs.est.6b04142.

(9) Hoch, A. R.; Reddy, M. M.; Aiken, G. R. Calcite Crystal Growth Inhibition by Humic Substances with Emphasis on Hydrophobic Acids from the Florida Everglades. *Geochim. Cosmochim. Acta* **2000**, *64* (1), 61–72. https://doi.org/10.1016/S0016-7037(99)00179-9.

(10) Khwaja, A. R.; Bloom, P. R.; Brezonik, P. L. Binding Constants of Divalent Mercury (Hg2+) in Soil Humic Acids and Soil Organic Matter. *Environ. Sci. Technol.* **2006**, *40* (3), 844–849. https://doi.org/10.1021/es051085c.

(11) Dong, W.; Liang, L.; Brooks, S.; Southworth, G.; Gu, B. Roles of Dissolved Organic Matter in the Speciation of Mercury and Methylmercury in a Contaminated Ecosystem in Oak Ridge, Tennessee. *Environ. Chem.* **2010**, *7* (1), 94. https://doi.org/10.1071/EN09091.

(12) Haitzer, M.; Aiken, G. R.; Ryan, J. N. Binding of Mercury(II) to Dissolved Organic Matter: The Role of the Mercury-to-DOM Concentration Ratio. *Environ. Sci. Technol.* **2002**, *36* (16), 3564–3570. https://doi.org/10.1021/es025699i.

(13) Mašek, O.; Buss, W.; Roy-Poirier, A.; Lowe, W.; Peters, C.; Brownsort, P.; Mignard, D.; Pritchard, C.; Sohi, S. Consistency of Biochar Properties over Time and Production Scales: A Characterisation of Standard Materials. *J. Anal. Appl. Pyrolysis* **2018**, *132*, 200–210. https://doi.org/10.1016/j.jaap.2018.02.020.

(14) Sigmund, G.; Hüffer, T.; Hofmann, T.; Kah, M. Biochar Total Surface Area and Total Pore Volume Determined by N2 and CO2 Physisorption Are Strongly Influenced by Degassing Temperature. *Sci. Total Environ.* **2017**, *580*, 770–775. https://doi.org/10.1016/j.scitotenv.2016.12.023.

(15) ASTM D 1762-84. Standard Test Method for Chemical Analysis of Wood Charcoal. In *ASTM International*; 2011; Vol. 84, pp 1–2. https://doi.org/10.1520/D1762-84R21.

(16) Sigmund, G.; Santín, C.; Pignitter, M.; Tepe, N.; Doerr, S. H.; Hofmann, T. Environmentally Persistent Free Radicals Are Ubiquitous in Wildfire Charcoals and Remain Stable for Years. *Commun. Earth Environ.* **2021**, *2* (1), 1–6. https://doi.org/10.1038/s43247-021-00138-2.

(17) Bloom, N. S.; Preus, E.; Katon, J.; Hiltner, M. Selective Extractions to Assess the Biogeochemically Relevant Fractionation of Inorganic Mercury in Sediments and Soils. *Anal. Chim. Acta* **2003**, *479* (2), 233–248. https://doi.org/10.1016/S0003-2670(02)01550-7.

(18) Webb, S. M. The MicroAnalysis Toolkit: X-Ray Fluorescence Image Processing Software. *AIP*

Conf. Proc. **2010**, *1365* (September 2011), 196–199. https://doi.org/10.1063/1.3625338.

(19) Auvray, P.; Genêt, F. Affinement de La Structure Cristalline Du Cinabre Α-HgS. *Bull. la Société française Minéralogie Cristallogr.* **1973**, *96* (3), 218–219. https://doi.org/10.3406/bulmi.1973.6816.

(20) Calos, N. J.; Kennard, C. H. L. K.; Davis, R. L. The Structure of Calomel, Hg2Cl2, Derived from Neutron Powder Data. *Zeitschrift fur Krist. - New Cryst. Struct.* **1989**, *187* (3–4), 305–307. https://doi.org/10.1524/zkri.1989.187.3-4.305.

(21) Bone, S. E.; Bargar, J. R.; Sposito, G. Mackinawite (FeS) Reduces Mercury(II) under Sulfidic Conditions. *Environ. Sci. Technol.* **2014**, *48* (18), 10681–10689. https://doi.org/10.1021/es501514r.

(22) Bone, S. E.; Bargar, J. R.; Sposito, G. Mackinawite (FeS) Reduces Mercury(II) under Sulfidic Conditions. *Environ. Sci. Technol.* **2014**, *48* (18), 10681–10689. https://doi.org/10.1021/es501514r.

(23) Manceau, A.; Nagy, K. L. Relationships between Hg(Ii)-S Bond Distance and Hg(Ii) Coordination in Thiolates. *Dalt. Trans.* **2008**, No. 11, 1421–1425. https://doi.org/10.1039/b718372k.

(24) Calvin, S. *XAFS for Everyone*, 1st ed.; CRC Press: Boca Raton, 2013. https://doi.org/10.1201/b14843.
